# Fibronectin promotes directional persistence in fibroblast migration through interactions with both its cell-binding and heparin-binding domains

**DOI:** 10.1038/s41598-017-03701-0

**Published:** 2017-06-16

**Authors:** Dimitris Missirlis, Tamás Haraszti, Horst Kessler, Joachim P. Spatz

**Affiliations:** 1 Department of Cellular Biophysics, Max Planck Institute for Medical Research, INF 253, D-69120 Heidelberg, Germany; 2 Department of Biophysical Chemistry, Heidelberg University, INF 253, D-69120 Heidelberg, Germany; 3 DWI – Leibniz Institute for Interactive Materials, Forckenbeckstr. 50, D-52056 Aachen, Germany; 4 Institute for Advanced Study and Center for Integrated Protein Science (CIPSM), Technische Universität München, Lichtenbergstr. 4, D-85747 Garching, Germany

## Abstract

The precise mechanisms through which insoluble, cell-adhesive ligands induce and regulate directional cell migration remain obscure. We recently demonstrated that elevated surface density of physically adsorbed plasma fibronectin (FN) promotes high directional persistence in fibroblast migration. While cell-FN association through integrins α_5_β_1_ and α_v_β_3_ was necessary, substrates that selectively engaged these integrins did not support the phenotype. We here show that high directional persistence necessitates a combination of the cell-binding and C-terminal heparin-binding domains of FN, but does not require the engagement of syndecan-4 or integrin α_4_β_1_. FN treatment with various fixation agents indicated that associated changes in fibroblast motility were due to biochemical changes, rather than alterations in its physical state. The nature of the coating determined the ability of fibroblasts to assemble endogenous or exogenous FN, while FN fibrillogenesis played a minor, but significant, role in regulating directionality. Interestingly, knockdown of cellular FN abolished cell motility altogether, demonstrating a requirement for intracellular processes in enabling fibroblast migration on FN. Lastly, kinase inhibition experiments revealed that regulation of cell speed and directional persistence are decoupled. Hence, we have identified factors that render full-length FN a promoter of directional migration and discuss the possible, relevant mechanisms.

## Introduction

Much of our knowledge on mesenchymal cell migration stems from *in vitro* studies on flat substrates, functionalized with adhesive ligands. Migrating cells initially polarize to establish a leading and a trailing edge, even in the absence of external gradients, in a process that depends on the type and concentration of displayed adhesive ligands^1, 2^. Subsequently, new adhesions stabilize the expanding leading edge, while adhesions at the trailing edge disassemble, in order to allow for cell body translocation^3^. In absence of external gradients in the soluble or insoluble environment, the polarization process occurs randomly, and subsequent migration does not have a preferred orientation^1^. Nevertheless, the nature and properties of adhesive substrates regulate the speed and directional persistence during such migration, through mechanisms that are still incompletely understood.

We recently showed that homogeneous fibronectin (FN) coatings promote fibroblast polarization and high directional persistence in fibroblast migration^4^. This phenotype required substrate engagement of both α_5_β_1_ and α_v_β_3_ integrins, the two major FN receptors. On the other hand, substrates that displayed selective α_5_β_1_ and α_v_β_3_ integrin antagonism did not promote a similar phenotype, suggesting the requirement for additional signals and/or associated mechanisms. The aim of the current study was to elucidate the mechanisms through which FN promotes directional migration *in vitro*.

Fibronectin is an essential extracellular matrix (ECM) glycoprotein, the knock-out of which is embryonically lethal in mice^5^. It constitutes a major component of the provisional, dynamic ECM, which is crucial in development, wound healing and angiogenesis^6, 7^. Plasma FN is secreted by hepatocytes and circulates in blood, while cellular FN, an alternatively spliced isoform containing specific extra-domains, is secreted and deposited in the ECM by various cell types, including fibroblasts. FN is secreted as a soluble dimer and has been shown to form insoluble fibers upon integrin-mediated cell surface binding and force application^6, 8, 9^. Beyond providing an adhesive structural support, recognized by a multitude of cell surface receptors, FN additionally forms a template upon which other ECM proteins and soluble growth factors can bind and assemble^6, 10^. While FN deposition is necessary during physiological remodeling processes, its abnormal accumulation and sustained presence within tissues has been associated with tumorigenesis, atherosclerosis and fibrosis, highlighting its potential role in disease initiation and/or progression^11–13^. For the above reasons, FN has been thoroughly studied and extensively used as coating for *in vitro* cell adhesion and migration research.

**Figure 1:**
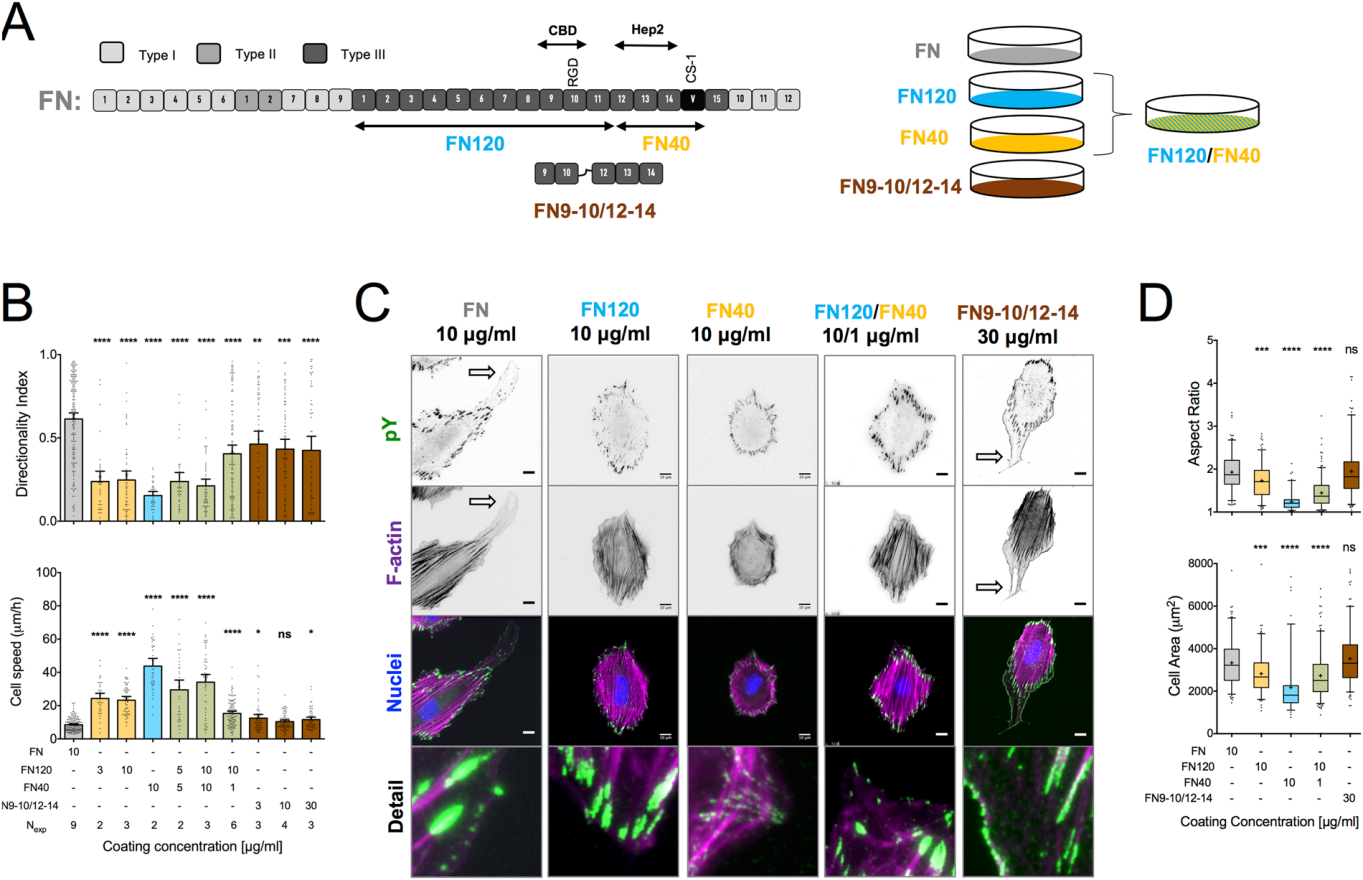
Signals from both the cell binding and C-terminal heparin-binding domains of FN are required for high directional persistence in fibroblast migration. (**A**) Schematic representation of plasma FN, showing the location of the different domains, the proteolytic fragments and recombinant fragments relevant for this study. Substrates were coated with FN, FN fragments or their combinations (color coded). (**B**) Cell speed and directionality index (distance from the origin divided by the trajectory length) were calculated for single REF migrating on indicated substrates for 16 hours. Cell speed was the lowest and directionality index was the highest on FN-coated substrates. N_exp_: number of independent experiments. Mean ± s.e.m. are presented. (**C**) Epifluorescence microscopy images of REF seeded for 6 hours on indicated substrates revealed important differences in adhesion cluster formation and F-actin cytoskeletal organization (see main text for details). Polarized protrusions are indicated by block arrows on FN and FN9–10/12–14. (**D**) REF projected cell area and aspect ratio, 6 hours post-seeding on substrates coated with FN or FN fragments. Results from >150 cells and 2 independent experiments are presented. The middle line in box plots indicates the median, the box indicates the interquartile range, the whiskers the 5^th^ and 95^th^ percentiles and the cross the mean. Scale bars: 10 μm. Data in (**B**,**D**) were compared to control FN-coated substrates using one-way ANOVA analysis. ns: not significant; *P < 0.05, **P < 0.01, ***P < 0.001, ****P < 0.0001.

The structure of FN is schematically shown in Fig. 1A: it consists of type I, type II and type III domains^14^. The major FN-binding integrin is α_5_β_1_, which recognizes the RGD binding site in the FNIII-10 domain and the synergy site PHRSN at the FNIII-9 domain^15^. However, several other integrins are known to bind FN^16^ and even compensate for its loss, in processes such as FN fibrillogenesis^17^.

In order to understand what drives high directional persistence in fibroblast migration on FN we studied the effects of 1) substrate-bound FN fragments and soluble blocking reagents to modulate ligand availability on the substrate and/or the cell surface, 2) small molecule inhibitors to interfere with intracellular signaling pathways, and 3) fixation agents to alter the physical state of FN on the substrate. We mostly avoided genetic manipulations, which could influence the migratory phenotype and did not add external directional cues, in order to examine the intrinsic propensity of fibroblasts for directional migration. Our overarching goal is to determine the necessary and sufficient signals that enable directional migration of cells.

## Results

### Directional persistence in fibroblast migration requires both the cell binding and heparin-binding 2 domains of FN

Selective engagement of α_5_β_1_ and α_v_β_3_ integrins on nano-patterned substrates was insufficient to recapitulate the high directional persistence in fibroblast migration observed on full-length FN^4^. In order to present the major FN integrin binding site in a more physiological manner, tissue culture polystyrene (TCPS) substrates were coated with the 120 kDa chymotryptic fragment of FN (FN120) containing the central cell-binding domain (CBD)^18, 19^. REF52 fibroblasts (REF) migrated faster, but with low directional persistence on FN120, compared to FN (Fig. 1B). The surface densities of FN120 tested were sufficient to support adhesion of REF at levels equal to FN (Supplemental Fig. S1A,B). REF on FN120 assembled focal adhesions (FAs) and aligned, ventral stress fibers similar to FN (Fig. 1C), but lacked polarized protrusions, which are defined as elongated protrusive regions free of stress fibers and rich in nascent adhesion at the cell edge (Fig. 1C and Supplemental Fig. S1E,F)^4^. Consequently, cells exhibited slightly reduced cell area and aspect ratio (Fig. 1D). These results indicated that fibroblast engagement to the CBD is insufficient to promote high directional persistence in fibroblast migration.

We hypothesized that additional interactions with the C-terminal region of FN are required to support high directional persistence^20, 21^. We therefore examined how co-adsorption of FN120 and the C-terminal, 40 kDa chymotryptic fragment (FN40), which contains the heparin-binding 2 domain (Hep2) and the connecting segment 1 (CS-1) of the variable region of FN (Fig. 1A), affected REF migration. On FN40-only substrates, cells adhered slower (Supplemental Fig. S1C), remained small and round (Fig. 1C and D), and exhibited high cell speed with erratic movements and low directionality (Fig. 1B). On substrates prepared with equal coating concentrations of FN40 and FN120, REF speed and directional persistence remained similar to the levels observed for FN120. Interestingly, mixing the two fragments at a 10:1 ratio (FN120:FN40) led to a reduction of cell speed and an increase of directionality index, albeit not to the levels attained on FN (Fig. 1B). Nonetheless, REF morphology on these substrates was similar to that on FN120 (Fig. 1C and D), with low aspect ratio and absence of polarized protrusions (Fig. 1C and S1F). These data indicate that polarized protrusions do not correlate with directional persistence, confirming our previous findings^4^.

The lack of control over the adsorbed amounts and ratio between the two individual fragments prompted us to use instead recombinant FN fragments that contain the CBD fused to the Hep2 domain and ensure a 1:1 molar ratio (FN9–10/12–14)^22, 23^. The directionality index for REF on substrates coated with FN9–10/12–14 was similar to that on FN120:FN40 (10:1 ratio), but still not as high as that for full-length FN (Fig. 1B). REF morphology was very similar on FN9–10/12–14 and FN (Fig. 1,C and D). Interestingly, a large fraction of cells exhibited polarized protrusions on FN9–10/12–14 (Supplemental Fig. S1F), pointing to the importance of appropriate spatial arrangement and/or ratio of presented ligands for their formation.

Overall, our data indicate that alone the CBD of FN does not promote directional persistence during fibroblast migration, while appropriate presentation of both the CBD and Hep2 domains on the substrate enhance directional persistence, albeit to a lower extent than full-length FN.

### Integrin α_4_β_1_ engagement to the Hep2 or CS-1 domains does not contribute to high directional persistence

**Figure 2:**
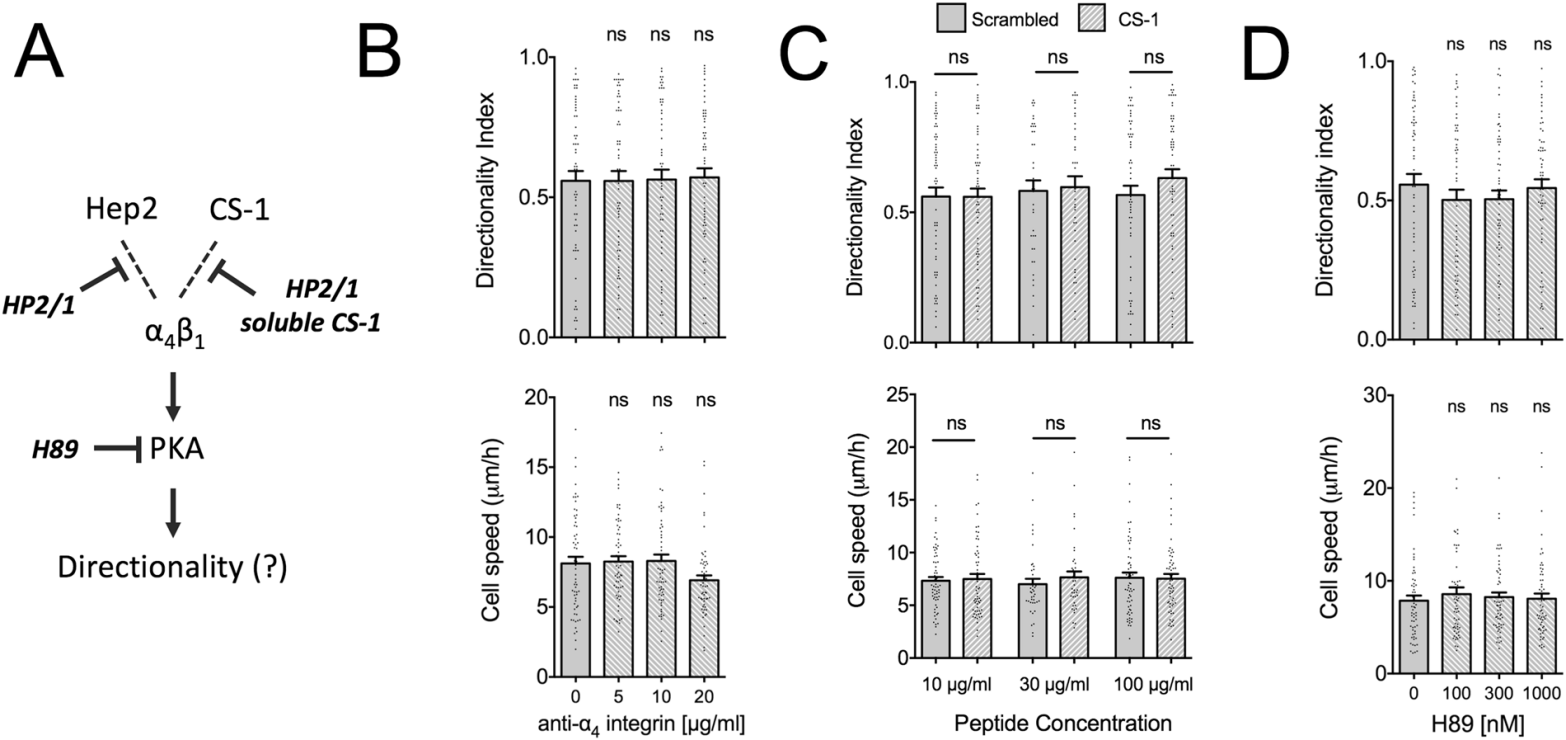
Integrin α_4_β_1_ blocking or PKA inhibition do not affect cell speed and directional persistence of fibroblast migration on FN. (**A**) Model of potential α_4_β_1_-mediated regulation of directional persistence in migration. Dashed lines indicate potential binding to integrin ligands and blocking reagents used are shown in bold. (**B**) REF speed and directionality index on FN-coated substrates (10 μg/ml) were not affected by the presence of blocking antibody against integrin α_4_β_1_ (clone H2P/1). Values in presence of antibody were compared to the control condition using one-way ANOVA analysis (N_exp_ = 3; n = 60). (**C**) REF speed and directionality index on FN-coated substrates (10 μg/ml) were not affected by the presence of CS-1 peptide, compared to a scrambled control. Values were compared using a Mann-Whitney test (N_exp_ = 3; n = 60 for peptide concentrations of 10 and 100 μg/ml and N_exp_ = 2; n = 40 for 30 μg/ml). (**D**) REF speed and directionality index on FN-coated substrates (10 μg/ml) were not affected by the presence of the soluble PKA inhibitor H89. Values in presence of H89 were compared to control conditions (same amount of DMSO as used for the highest H89 concentration) using one-way ANOVA analysis (N_exp_ = 3; n = 57–60). Mean ± s.e.m. are presented. ns: not significant.

Our results point to the possibility that additional cell receptor-FN interactions at its C-terminal region are necessary for promoting high directional persistence. Integrin α_4_β_1_ binds to both Hep2 and CS-1 and was shown to regulate leading edge advancement and polarization^24^. In particular, protein kinase A (PKA)-mediated phosphorylation of α_4_ integrin at the leading edge was suggested to promote directional migration^25, 26^. Blocking of α_4_β_1_ integrin binding, using either blocking antibodies against α_4_ (clone HP2/1) or soluble CS-1 peptides, did not affect cell speed or directional persistence on FN (Fig. 2A–C). Moreover, PKA inhibition with the inhibitor H89, at concentrations, which result in efficient inhibition^27^, had no effect on migration speed or directional persistence (Fig. 2D). At higher concentrations, H89 inhibits many kinases, including Rho kinase^28^, inhibition of which causes an increase in cell speed and decrease in directional persistence^4^. Accordingly, H89 at concentrations above 10 μM resulted in loss of stress fibers, higher cell speed and reduced directional persistence, most likely due to off-target effects of H89 (Supplemental Fig. S2). Taken together, our results suggest that engagement of FN by α_4_β_1_ integrin and PKA-mediated signaling do not contribute to the high directional persistence in fibroblast migration observed on FN.

### Syndecan-4 engagement does not contribute to high directional persistence in fibroblast migration

**Figure 3:**
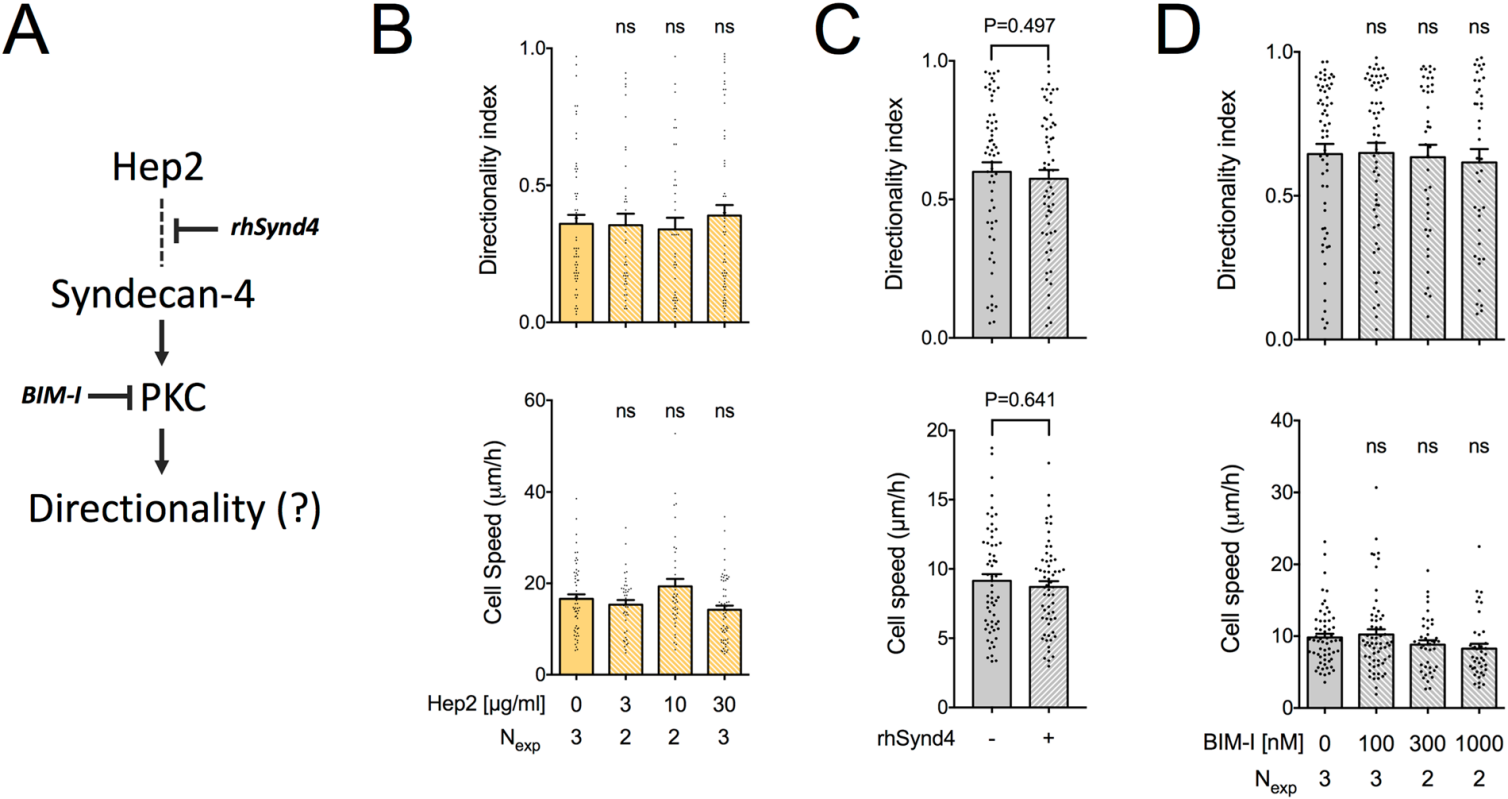
Syndecan-4 engagement and PKC activation are not required for high directional persistence on FN. (**A**) Model of potential syndecan-4-mediated regulation of directional persistence in migration. (**B**) REF speed and directionality index on FN120-coated substrates (10 μg/ml) were not affected by the presence of soluble Hep2 fragments. Values in presence of Hep2 were compared to the control condition using one-way ANOVA analysis. (**C**) REF speed and directionality index on FN-coated substrates (10 μg/ml) were not affected by the presence of a recombinant syndecan-4 ectodomain (10 μg/ml). Values were compared using a Mann-Whitney test (N_exp_ = 3; n = 60). (**D**) REF speed and directionality index on FN-coated substrates (10 μg/ml) were not affected by the presence of the soluble PKC inhibitor bisindolylmaleimide I (BIM-I). Values in presence of BIM-I were compared to control conditions (same amount of DMSO as used for the highest inhibitor concentration) using one-way ANOVA analysis. N_exp_: number of independent experiments. Mean ± s.e.m. are presented. ns: not significant.

The Hep2 domain is additionally a ligand for syndecan-4, a surface proteoglycan suggested to promote directional migration through regulation of small GTPase signaling (Fig. 3A)^29^. It was previously shown that providing ligands for syndecan-4, via addition of soluble Hep2 fragments to cells attached on the CBD, was sufficient to rescue small GTPase activation^30, 31^, FA formation^20, 32^ and directional migration^30^. However, incubation with soluble Hep2 fragments, even at concentrations exceeding those previously used, did not significantly enhance directional persistence of REF migrating on FN120 in our experimental setup (Fig. 3B). Moreover, competition of recombinant syndecan-4 ectodomain (rhSynd4) with membrane-bound syndecan-4 for FN-binding sites did not cause a noticeable change in motility of REF on FN (Fig. 3C).

Syndecan-4 was shown to promote directional migration following protein kinase C (PKC) activation and subsequent redistribution of activated p190RhoGAP to regulate FA formation and protrusion dynamics^20, 30, 31, 33^. PKC inhibition using bisindolylmaleimide I (BIM-1), at concentrations previously shown to be both effective and selective^31, 34^, had no effect on REF cell speed or directional persistence on FN (Fig. 3D). This finding further argues against the requirement of CD44 binding to the Hep2 domain for directional migration as previously proposed, since CD44 also acts through PKC^35^. In summary, our results suggest that syndecan-4 engagement and PKC-mediated signaling do not contribute to the high directional persistence in fibroblast migration observed on FN.

### Soluble Hep2 does not reduce directional persistence on FN

**Figure 4:**
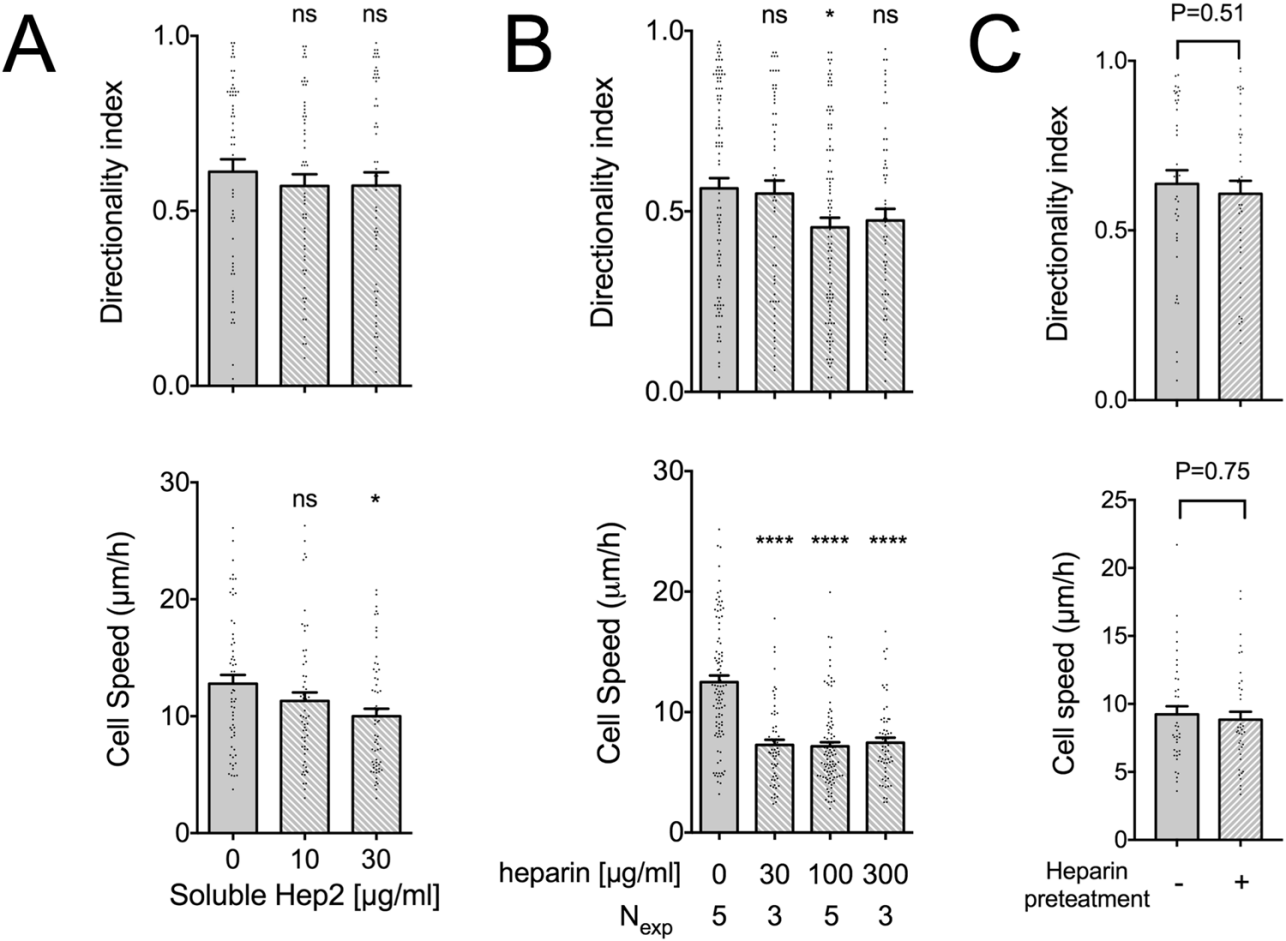
Soluble heparin reduces fibroblast speed on FN but has only a mild effect on directional persistence. (**A**) REF speed on FN-coated substrates (10 μg/ml) slightly decreased with increasing concentrations of soluble Hep2 domain. Directional persistence on the other hand did not change. Values in presence of Hep2 were compared to the control condition using one-way ANOVA analysis (N_exp_ = 3; n = 60). (**B**) REF speed markedly decreased in presence of soluble heparin on FN-coated substrates (10 μg/ml), whereas directionality index showed a significant decrease at a heparin concentration of 100 μg/ml, but not at 300 μg/ml. Values in presence of heparin were compared to the control condition using one-way ANOVA analysis. N_exp_: number of independent experiments. Mean ± s.e.m. are presented. ns: not significant, *P < 0.05, ****P < 0.0001. (**C**) FN-coated TCPS substrates (10 μg/ml) were pre-incubated with 100 μg/ml heparin for 15 minutes at 37 °C, washed 3 times with PBS and seeded with REF. Cell speed and directionality index did not show significant differences compared to untreated FN-coated substrates. Mean ± s.e.m. are presented (n = 40 cells; N_exp_ = 2). Values were compared using Mann-Whitney tests.

The ability of co-adsorbed Hep2 to enhance directional persistence on the CBD, led us to examine whether incubation with *soluble* Hep2 would impact fibroblast motility on full-length FN. Soluble Hep2 had no significant effect on directional persistence, and showed a small decrease in cell speed at the highest concentration tested (Fig. 4A). These results suggest that soluble Hep2 does not inhibit directional persistence by competing for binding with a potential cell surface receptor, further supporting our findings that integrin α_4_β_1_ and syndecan-4 do not regulate directional persistence under our experimental conditions. It appears that Hep2 promotes directional persistence only when it is substrate bound (Fig. 1B).

We considered the possibility that adsorbed Hep2 acts by recruiting soluble factors to the substrate^22, 36^, thus enabling efficient cross-talk with integrins bound to the CBD. We hypothesized that heparin might compete with the binding of such factors and/or of surface proteoglycans to the Hep2 domain of FN^37, 38^. Incubation with soluble heparin markedly reduced REF speed as previously reported^38–40^, and induced a modest, yet statistically significant decrease in directional persistence for a heparin concentration of 100 μg/ml, but not of 300 μg/ml (Fig. 4B). Heparin was also reported to reversibly bind and catalytically alter the conformation of substrate-adsorbed FN in a non-reversible manner^41–43^; importantly, previous studies showed that heparin pretreatment of FN increased availability of the Hep2 domain towards antibodies^44^ and growth factors^43, 45^. In our hands, pretreatment of FN with soluble heparin prior to REF seeding did not affect cell speed or directional persistence (Fig. 4C). This finding suggests that heparin does not influence migration speed through changes in FN conformation, but needs to be present in the medium to be effective^38^, perhaps by acting intracellularly as previously suggested^46^. Interestingly, a closer look at the effects of soluble Hep2 and heparin on adhesion cluster formation revealed differences: a considerable reduction in the fraction of cells presenting polarized protrusions was noted in presence of soluble heparin, but not of soluble Hep2 (Supplemental Fig. S1F), indicating distinct mechanisms of action.

### Fibronectin cross-linking impairs directional migration

**Figure 5:**
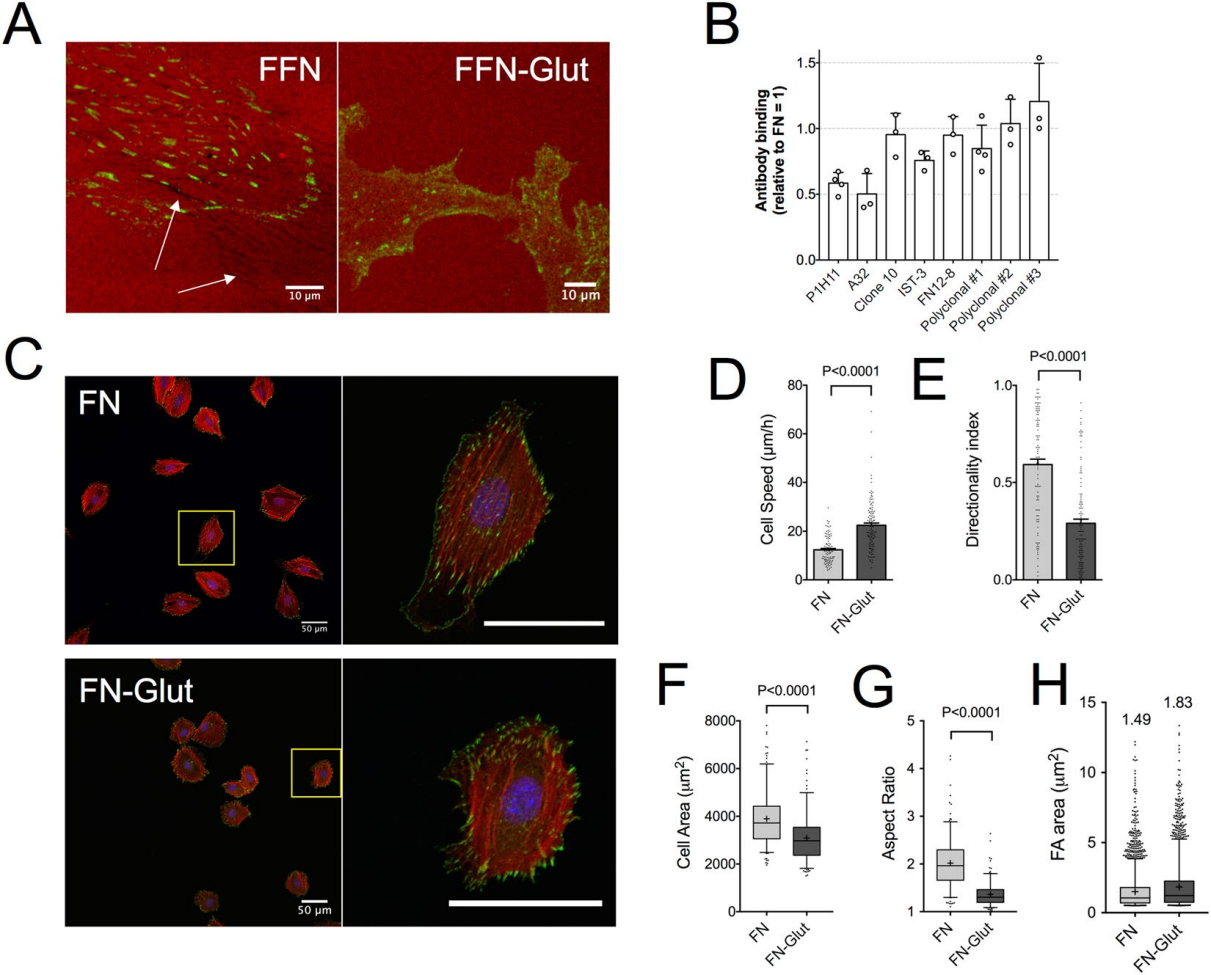
FN cross-linking with glutaraldehyde hinders fibroblast polarization and reduces directional persistence in fibroblast migration. (**A**) Confocal microscopy images of REF, fixed 24 hours after seeding on control substrates coated with 10 μg/ml fluorescently-labeled FN (red; FFN) or treated with 1% glutaraldehyde (FFN-Glut). Cells were immunostained against tensin-1 (green). Arrows indicate streak-like patterns where FFN was removed (remodeled) from the substrate in the absence of glutaraldehyde cross-linking. (**B**) Quantification of antibody binding to FN-Glut using a modified ELISA assay showed reduced binding compared to FN-coated substrates of some monoclonal anti-FN antibodies (clones P1H11, A32 and IST-3), indicating altered FN conformation upon glutaraldehyde cross-linking. On the other hand, all tested polyclonal antibodies and some of the monoclonal antibodies (clones 10 and FN12-8) displayed similar binding efficiency to FN. Each point represents an independent experiment with n =﻿ 2–5. Mean ± s.d. are presented. (**C**) Confocal microscopy images of REF on FN-Glut or FN-coated substrates, fixed 6 hours after seeding and stained against F-actin (red), pY to indicate cell-substrate adhesions (green) and DNA to image the nucleus (blue). Images on the right are zoomed views of the highlighted yellow boxes. Scale bars 50 μm. (**D**) REF speed increased on FN-Glut, while (**E**) directionality index was markedly reduced compared to FN-coated substrates (N_exp_ = 3; n = 60). Mean ± s.e.m. are presented in (**D**,**E**). (**F**) REF projected area and (**G**) aspect ratio were lower on FN-Glut substrates (>200 cells analyzed; N_exp_ = 2). (**H**) FA area increased on FN-Glut compared to FN-coated substrates (mean values indicated on graph; >2500 FAs analyzed; N_exp_ = 2). The middle line in box plots indicates the median, the box indicates the interquartile range, the whiskers the 5^th^ and 95^th^ percentiles and the cross the mean. Values were compared using an unpaired t-test with Welch’s correction.

Our data so far indicate that a direct interaction between cell surface receptors and Hep2 is not necessary for high directional persistence on FN. We considered alternative, physical mechanisms, and in particular, whether the physical state of the coating could be responsible for the substrate dependence in regulating directional persistence of migration. One possibility is that FN stretching, remodeling and polarized fibril assembly under migrating cells functions as a physical guidance cue, creating a positive feedback loop to maintain directionality. As a first, rough approach to test our hypothesis, we treated FN with glutaraldehyde to induce inter- and intramolecular cross-links, expected to inhibit its remodeling. Of note, substrates were extensively washed and possible remaining reactive groups were blocked with bovine serum albumin (BSA). Using fluorescently-labeled FN, we confirmed that REF were unable to remodel glutaraldehyde-treated FN, whereas FN was removed in streak-like patterns under REF migrating on adsorbed FN (Fig. 5A and Supplemental Fig. S3A).

Glutaraldehyde treatment of adsorbed FN (FN-Glut) did not alter its overall amount on the substrate (Supplemental Fig. S3B); however, we measured differences in antibody binding efficiency between FN and FN-Glut, suggesting either changes in FN conformation that mask the binding sites, or the destruction of epitopes as a result of the cross-linking reaction (Fig. 5B). In particular, we noted a decrease in binding of monoclonal antibodies against the CBD (clone P1H11) and Hep2 domains (clone Ab32) on FN-Glut, but not of polyclonal antibodies (Fig. 5B). Despite the decrease in accessibility to the CBD, REF adhered on FN-Glut with the same efficiency compared to FN (Supplemental Fig. S3C). Selective blocking of α_5_β_1_ or α_v_β_3_ integrins using small molecule antagonists^4, 47^ revealed that REF bind primarily through α_5_β_1_ integrins to FN and FN-Glut (Supplemental Fig. S3C). Interestingly, REF adhesion to FN-Glut was more sensitive to α_5_β_1_ integrin blocking, compared to FN (Supplemental Fig. S3C). The increased sensitivity on FN-Glut of over one order of magnitude in concentration of α_5_β_1_ integrin antagonists is unlikely to be due to a reduction in the number of binding sites by half (Fig. 5B); instead, this result suggests that REF interact with non-modified FN using additional receptors.

Motility assays on FN-Glut showed a pronounced decrease in directional persistence, and an increase in cell speed compared to FN (Fig. 5D and E). REF seeded on FN-Glut exhibited lower spread area and aspect ratio compared to FN (Fig. 5C,F and G), with cells rarely forming polarized protrusions (Supplemental Fig. S1F). FAs were slightly larger on FN-Glut (Fig. 5H), indicating stronger association with the substrate. Importantly, formation of fibrillar adhesions was blocked on FN-Glut, as evidenced by the lack of elongated tensin-1 and cell-secreted FN under the cells (Supplemental Fig. S3A), consistent with previous reports^48, 49^. Taken together, our results demonstrate that glutaraldehyde cross-linking of adsorbed FN impairs fibroblast polarization and hinders directional persistence in migration.

### Directional persistence correlates with FN ligand availability and not FN physical state

We next tested the effect of treating FN with two additional fixation agents on REF motility. Paraformaldehyde (PFA) is a milder cross-linking agent compared to glutaraldehyde^50^, inducing predominantly intramolecular bonds, while methanol (MeOH) fixes FN via dehydration-induced precipitation. In addition, we tested an increased glutaraldehyde concentration of 5%. Imaging of fluorescently-labeled FN confirmed the lack of remodeling following PFA or MeOH addition (Supplemental Fig. S4A).

**Figure 6:**
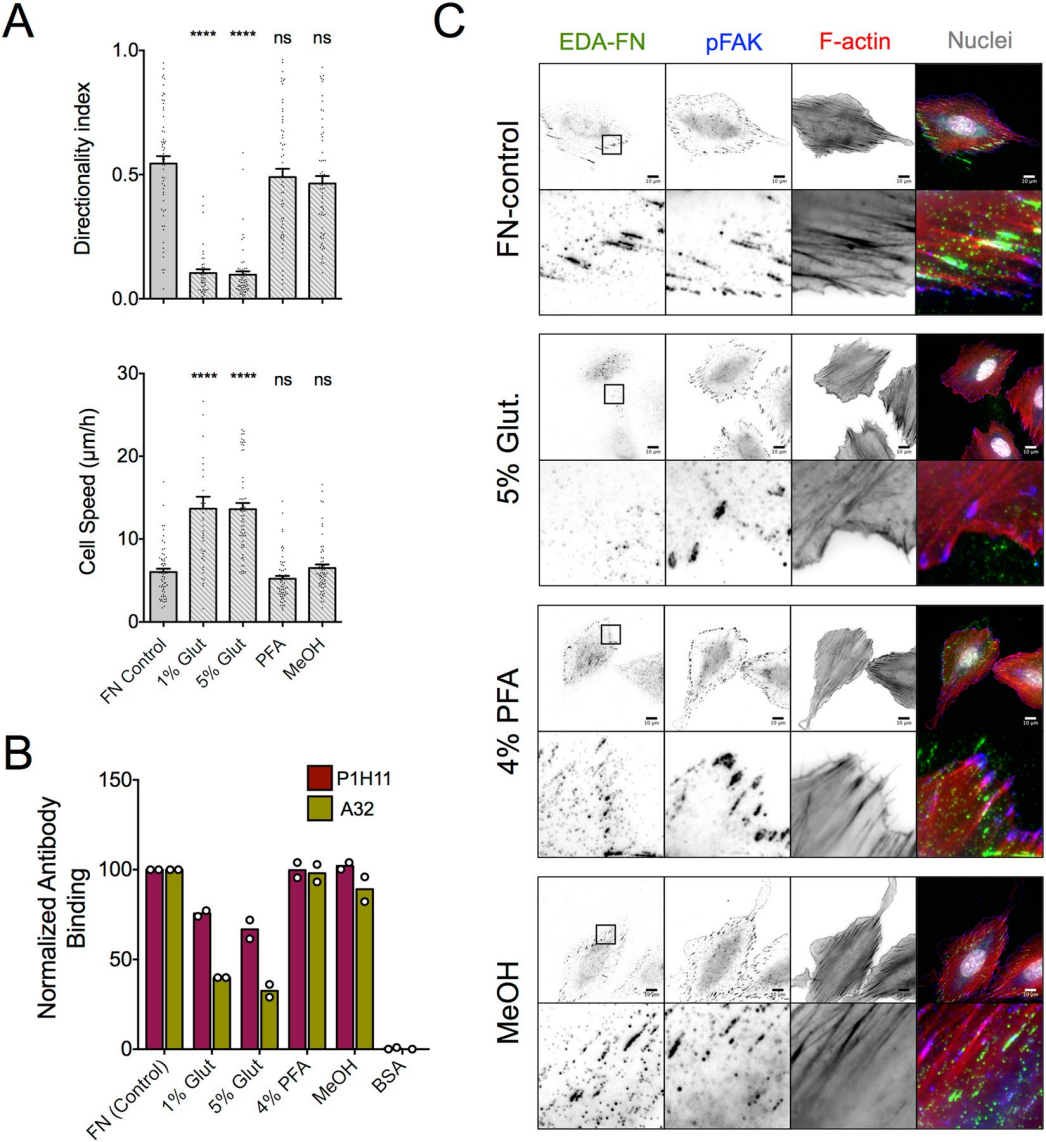
REF motility dependence upon FN treatment with different fixation agents correlates with induced changes in ligand accessibility. (**A**) REF speed and directionality index on FN-coated substrates (10 μg/ml) treated with different fixation agents. Cell speed and directional persistence were affected by glutaraldehyde (Glut), but not PFA or MeOH treatment of FN prior to cell seeding. Values from treated samples were compared to the control condition using one-way ANOVA analysis (N_exp_ = 2; n = 40 cells for 1% Glut, N_exp_ = 3, n = 60 cells for other samples). Mean ± s.e.m. are presented. ns: not significant, ****P < 0.0001. (**B**) Quantification of antibody binding to FN-coated (control) or FN-treated substrates using a modified ELISA assay. Reduced binding of monoclonal anti-FN antibodies against the CBD (clone P1H11) and the Hep2 domain (clone A32) was observed only for glutaraldehyde-treated samples. Each point represents an independent experiment (n = 5 samples/independent experiment). (**C**) Epifluorescence microscopy images of REF seeded for 6 hours on FN-coated substrates, fixed and stained against indicated proteins. REF were able to form polarized protrusions and assemble cellular FN (EDA-FN) on control, PFA-treated and MeOH-treated substrates, but not glutaraldehyde-treated ones.

REF exhibited similar speed and directional persistence on FN-coated, PFA-treated and MeOH treated substrates (Fig. 6A). Again, glutaraldehyde treatment resulted in a pronounced increase of cell speed and decrease in directional persistence, with no apparent dependence on glutaraldehyde concentration (Fig. 6A). REF adhered with similar efficiency on FN control and treated surfaces (Supplemental Fig. S4B). On the other hand, accessibility to the CBD and Hep2 domain, as assessed by monoclonal antibody binding, was impaired only on glutaraldehyde-treated substrates, but not PFA- or MeOH-treated ones (Fig. 6B). Additionally, REF exhibited similar morphology, formed polarized protrusions and assembled cellular FN, containing the alternatively spliced domain A (EDA-FN), on control, PFA- and MeOH-treated substrates, but not glutaraldehyde-treated ones (Fig. 6C). These results suggest that glutaraldehyde cross-linking impairs fibroblast polarization and directional persistence in migration primarily due to alterations in ligand accessibility, rather than in FN physical state.

### Inhibition of FN fibrillogenesis reduces directional persistence in migration

**Figure 7:**
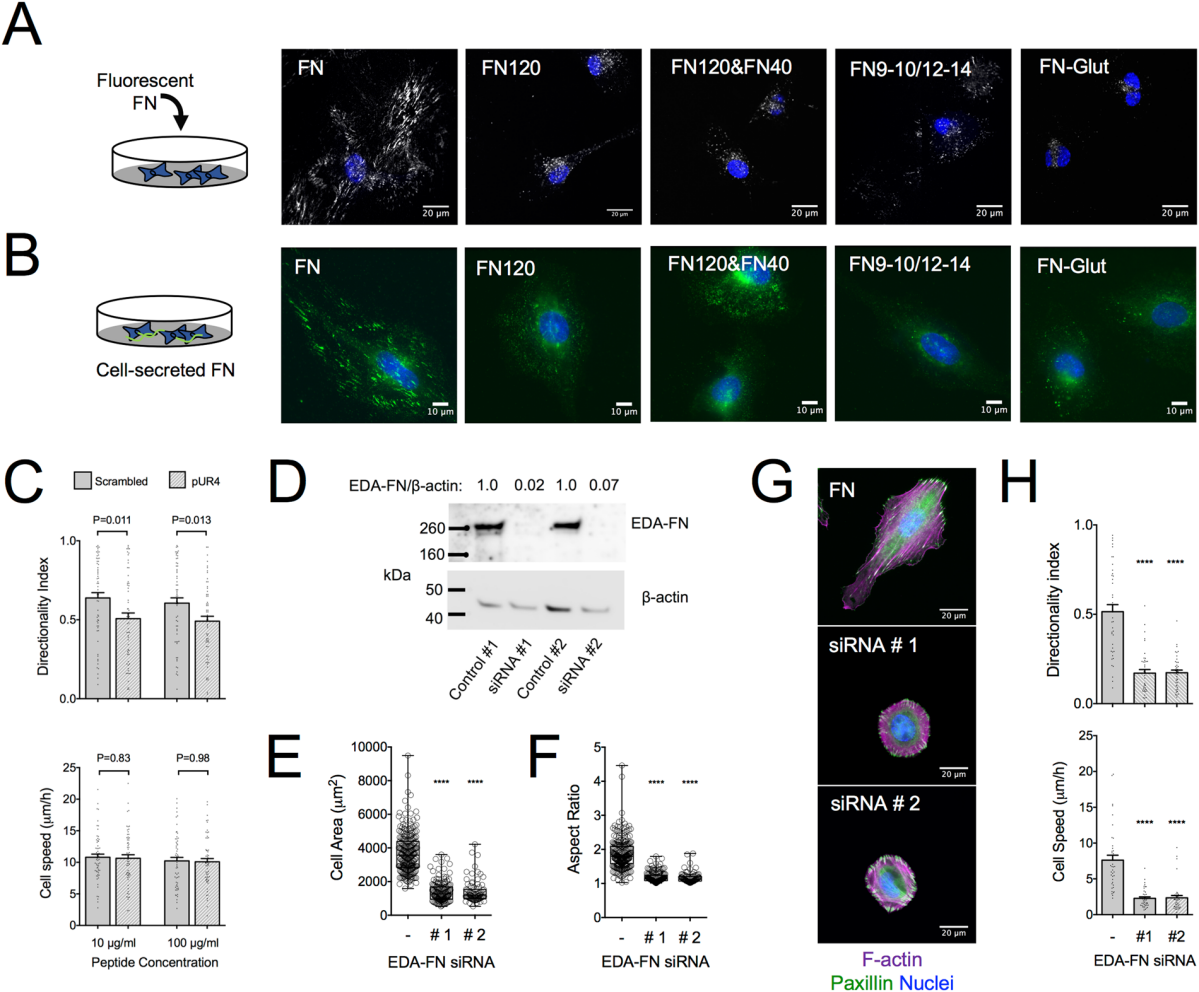
FN fibrillogenesis inhibition impairs directional persistence in fibroblast migration and cellular FN knockdown abolishes migration altogether. (**A**) Confocal microscopy images of fluorescently-labeled FN (gray) bound and/or assembled by REF on substrates with indicated coatings. Culture medium was exchanged 4 hours after seeding with medium containing 10 μg/ml FFN and incubated for another 20 hours, prior to washing and cell fixation. Fibrillogenesis of soluble FFN was observed only on FN-coated substrates. Nuclei are stained with DAPI (blue). (**B**) Epifluorescence microscopy images of REF immunostained against cellular FN (green), fixed 24 hours after seeding on substrates with indicated coatings. FN fibrillogenesis was evident only on FN-coated substrates. Nuclei are stained with DAPI (blue). Coating concentrations of FN: 10 μg/ml, FN120: 10 μg/ml, FN120/FN40: 10/1 μg/ml and FN9–10/12–14: 10 μg/ml. (**C**) REF directionality index on FN-coated substrates was reduced in presence of pUR4 peptide compared to a scrambled control peptide, whereas cell speed was unaffected. Values were compared using a Mann-Whitney test (N_exp_ = 3; n = 60). Mean ± s.e.m. are presented. (**D**) Western blot analysis of lysates from REF treated 48 hours with siRNA against EDA-FN. The EDA-FN/β-actin ratio was obtained by densitometric analysis. (**E**) REF projected cell area and (**F**) aspect ratio 6 hours post-seeding on substrates coated with FN. EDA-FN knockdown resulted in a large reduction of cell area and cell rounding (N_exp_ = 2). Values of siRNA-treated REF were compared to the control condition using one-way ANOVA analysis (****P < 0.0001). (**G**) Epifluorescence microscopy images of control or siRNA-treated REF seeded for 6 hours on FN-coated substrates, fixed and stained against paxillin (green), F-actin (magenta) and DNA (blue). EDA-FN knockdown changed dramatically cell morphology but did not impair focal adhesion or stress fiber formation. (**H**) REF speed and directionality index calculated for control and siRNA-treated REF on FN. EDA-FN knockdown abolished migration, with residual cell speed reflecting nuclei movements within the cell body. Data were compared using one way ANOVA analysis (****P < 0.0001). Mean ± s.e.m. are presented (N_exp_ = 2; n = 40).

An additional, possible contributing factor to the loss of directional persistence on FN-Glut is the consequent inhibition of FN fibrillogenesis (Supplemental Fig. S3A). REF were able to assemble fibrillar structures of both endogenous (EDA-FN) and externally-added fluorescent FN, on FN-coated substrates, but not on FN-Glut, FN120, FN120 and FN40, or FN9–10/12–14 (Fig. 7A and B). Thus, substrate coating has an impact on FN fibrillogenesis, consistent with previous reports^51, 52^. The fact that FN fibrillogenesis was impaired on FN-Glut prompted us to investigate whether pharmacological inhibition of fibrillogenesis leads to loss of directional persistence on FN. Incubation with the pUR4 peptide^53^ inhibited FN fibrillogenesis on 3T3 fibroblasts that expresses fluorescent FN (3T3-FN), verifying pUR4 activity (Supplemental Fig. S5). Incubating REF with 10 μg/ml pUR4 resulted in a small, but significant reduction of directional persistence compared to the scrambled control peptide, without a change in cell speed (Fig. 7C). Even at higher pUR4 concentrations (100 μg/ml), the decrease in directional persistence was modest, compared to that observed on FN-Glut. Together, our results suggest that FN fibrillogenesis contributes to high directional persistence in fibroblast migration to a low, yet significant degree, and thus it is not the principal factor responsible for the low directionality observed on FN-Glut.

### Cell-secreted FN is essential for fibroblast migration

We next examined the contribution of FN present in the culture medium on directional migration. REF motility on FN-coated substrates, in absence of soluble FN in the medium, was indistinguishable from control conditions (Supplemental Fig. S6). Thus, the presence of soluble FN is dispensable for high directional persistence in fibroblast migration on FN. In contrast, siRNA knock-down of EDA-FN had a pronounced effect on fibroblast motility. Two different siRNA sequences, which led to >90% silencing of EDA-FN (Fig. 7D), prevented REF polarization, without inhibiting FA or stress fiber formation (Fig. 7E–G). Intriguingly, REF motility was abolished (Video 1), with the calculated cell speed reflecting mainly nuclear movements within immotile cells (Fig. 7H). Inhibition of total protein synthesis using cycloheximide resulted in a pronounced decrease of expressed EDA-FN, and had a similar effect on cell motility (Supplemental Fig. S7). These results demonstrate that EDA-FN expression is necessary for chemokinesis on FN-coated substrates, but preclude conclusions on the role of cell-secreted FN in regulating directionality.

### Involvement of signaling pathways in fibroblast migration

**Figure 8:**
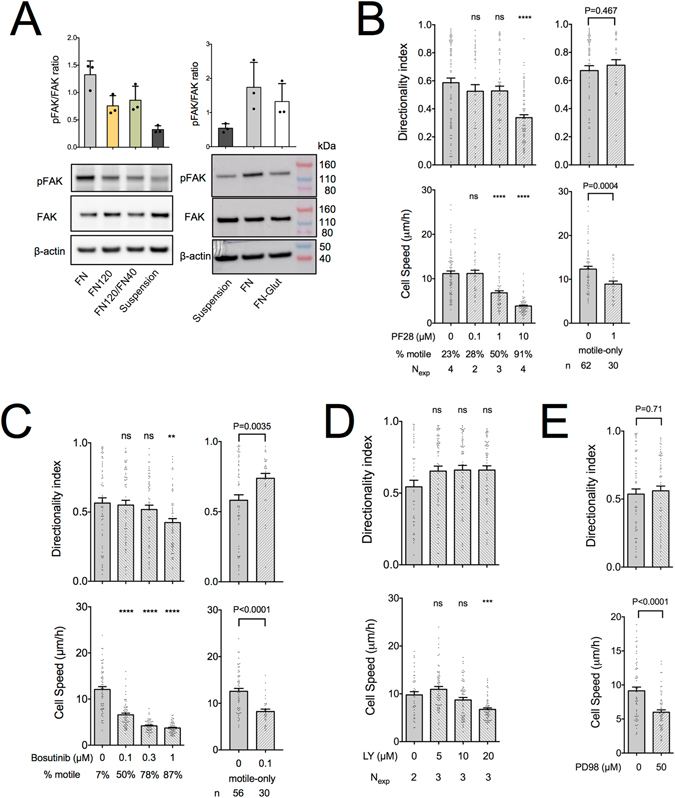
Cell speed and directional persistence in fibroblast migration are differentially regulated. (**A**) Western blot analysis of phosphorylated (Y397) and total FAK from REF lysates, prepared from cells seeded on indicated substrates for 30 minutes. Quantification through densitometry (N_exp_ = 3) revealed higher pFAK_Y397_ levels on FN compared to FN120, FN120/FN40 and FN-Glut. Mean ± s.d. are presented. (**B**) REF speed and directionality index on FN in presence of the FAK inhibitor PF-573228 (PF28). 1 μM PF28 significantly reduced cell speed but did not affect directional persistence. At 10 μM, cells did not translocate, but the nucleus still wiggled within the cell body, giving rise to a low speed and directionality index. The percentage of motile cells (cells exhibiting a maximum displacement of >50 μm from the point of origin at any time during the observation period) and the number of independent experiments are indicated on the graph. When motile-only cells were analyzed, a non-significant increase in directionality index was observed for cells treated with 1 μM PF28 compared to controls (unpaired t-test with Welch’s correction). (**C**) REF speed and directionality index on FN in presence of the c-Src inhibitor Bosutinib revealed similar results to FAK inhibition. At concentrations of 0.1 and 0.3 μM, Bosutinib significantly reduced cell speed but did not affect directional persistence. The percentage of motile cells is indicated on the graph (N_exp_ = 3; n = 60). Directionality index for motile-only cells was significantly higher for 0.1 μM Bosutinib compared to controls (unpaired t-test with Welch’s correction). (**D**) REF speed and directionality index on FN in presence of the PI3K inhibitor LY294,002 (LY). PI3K inhibition resulted in a reduction of cell speed at 20 μM, and a small, non-significant increase in directionality index. (**E**) REF speed and directionality index on FN in presence of the MEK inhibitor PD98059 (PD98). Inhibition of the ERK/MAPK pathway resulted in a significant reduction of cell speed but had no effect on directional persistence (N_exp_ = 3; n = 60). Control conditions (0 μM) in (**B**–**E**) correspond to samples with the same amount of DMSO as that used for the highest inhibitor concentration. Values in presence of inhibitors were compared to controls using one-way ANOVA analysis (**B**–**D**) or unpaired t-tests (**E**). Coating concentrations of FN: 10 μg/ml. Mean ± s.e.m. are presented. ns: not significant; **P < 0.01, ***P < 0.001, ****P < 0.0001.

In order to gain a mechanistic understanding of how differential substrate adhesion regulates fibroblast motility, and in particular directionality, we next investigated the involvement of some relevant intracellular signaling pathways. Our previous work suggested focal adhesion kinase (FAK) activation downstream of β_1_ integrin engagement as a promoter of directional migration^4^. FAK Y397 phosphorylation levels, indicative of FAK activation, were higher for REF on FN compared to FN120 or FN-Glut (Fig. 8A). However, pFAK levels were not enhanced on substrates coated with FN120:FN40 (ratio 10:1) compared to FN120 (Fig. 8A), despite higher directional persistence observed on the former coating (Fig. 1B).

FAK inhibition using high concentrations of PF-573228 (PF28) impedes migration, consistent with its role in regulating FA turnover and motility^4, 54–56^. However, by titrating PF28, we identified concentrations that significantly reduced the fraction of motile cells and their corresponding speed, but maintained high directional persistence (Fig. 8B). These data suggest that the level of FAK activation correlates with cell speed but not directional persistence.

c-Src kinase can complex with FAK to phosphorylate several effectors previously linked to cell migration, but may also exhibit FAK-independent action^57, 58^. c-Src inhibition using Bosutinib^59^ showed similar effects compared to FAK inhibition, i.e. loss of motility at high inhibitor concentrations and maintenance of high directional persistence at concentrations that still significantly reduced cell speed (Fig. 8C). Therefore, c-Src does not appear to be necessary for maintaining high directional persistence on FN.

Phosphatidylinositol 4,5-bisphosphate 3-kinase (PI3K) activation is linked to cell protrusion formation and persistence, as well as to directional migration during chemotaxis^60, 61^. PI3K inhibition using LY 294,002 (LY) resulted in a slightly positive, but statistically non-significant, effect on directional persistence and a decrease in cell speed, consistent with previous reports (Fig. 8D)^60, 62^. Interestingly, analysis of motile-only cells following mild c-Src or FAK inhibition, which act upstream of PI3K, showed a slight increase in directional persistence compared to controls (Fig. 8B and C). These data indicate that elevated PI3K activity is not necessary for high directional persistence on FN during chemokinesis, but instead appears to exert a negative effect in this respect.

We finally considered the involvement of mitogen-activated protein (MAP) kinase signaling in directional migration^63–66^. MAP kinase kinase (MEK) inhibition using PD98059 (PD98) resulted in a reduction of cell speed as previously reported^63, 65^, but had no effect on directional persistence (Fig. 8E).

Overall, the tested kinase inhibitors did not impair directional persistence in fibroblast migration, at concentrations which significantly reduced cell speed. This finding suggests that the associated intracellular pathways were not responsible for regulating directional migration during serum-stimulated chemokinesis, and that distinct mechanisms regulate cell speed and directional persistence during fibroblast migration.

## Discussion

In this study, we identified a set of necessary elements for promoting long-term directional persistence during fibroblast chemokinesis on homogeneously-coated FN. Availability of binding sites on both the CBD and C-terminal Hep2 domains of FN was necessary for promoting high directional persistence (Fig. 1B). Accordingly, the reduction in ligand accessibility upon glutaraldehyde cross-linking, due to epitope modification or masking, had a negative impact on directionality (Fig. 5). Interestingly, the amount and presentation of substrate-bound Hep2 relative to the CBD had to be tuned to induce directional persistence. This is in line with several reports showing that it is often less efficient to obtain a biological effect by combining FN fragments compared to forcing their stoichiometry using recombinant constructs^22, 67^. Future work with variable linkers between the CBD and Hep2 domains could aid in determining how their spatial proximity has a functional role in migration.

While the exact mechanism through which Hep2 contributes to an increase in directional persistence was not identified, our results nevertheless excluded several possibilities and bring forward certain hypotheses. Receptors and mechanisms, previously proposed to modulate directional migration via Hep2 association, were shown to be dispensable, under our experimental conditions. *Soluble* Hep2 did not impair directional migration on FN by competing for potential receptor binding. Moreover, neither integrin α_4_β_1_ nor syndecan-4, which signal through Hep2 binding, were found to be necessary for maintenance of high directional persistence during REF migration, despite their proposed roles in regulating directionality^24, 30^. Several studies demonstrating a requirement for syndecan-4 in cell spreading^68^, FA formation^69, 70^, PKC activation and small GTPase regulation^30, 31, 33, 71^, did not use serum in their experiments. Thus, the presence of serum in our study could have masked the effects of syndecan-4 engagement, as previously also shown^33, 69, 72^. Moreover, studies that did include serum and demonstrated a requirement for syndecan-4 were performed on aligned, cell-derived FN matrices, which differ over the physisorbed monolayers used here^30, 71^. Indeed, a report from the same group, which showed syndecan-4 requirement in directional migration^30^, reported no difference in directional persistence between syndecan-null and wild type fibroblasts in a single cell motility assay on physisorbed, globular FN^30, 73^. Therefore, it appears that the presentation and conformation of FN dictate the requirements for promoting directional cell migration.

One potential mechanism linking substrate-bound Hep2 and directionality could involve sequestering of relevant factors from the soluble milieu onto surface-bound Hep2 to regulate migration^22^. The ability of the Hep2 domain of FN to modulate the activity of various growth factors is well established^67, 74, 75^; furthermore, recent work demonstrated that control over FN conformation and fibrillar formation to expose Hep2, results in enhanced growth factor activity *in vivo* ^76^. It is therefore foreseeable that synergistic cross-talk between integrins and growth factor receptors recognizing substrate bound ligands^10, 77^ lies behind the regulation of directional persistence. Further experiments with defined culture media and growth factor composition would be required to examine this possibility.

Our combined results on the coating-dependence and impact of FN cross-linking on cell migration indicate the important role of FN conformation on regulating migration, and consequently bring forward considerations for the design and interpretation of *in vitro* studies. Altering the physical state of adsorbed FN through glutaraldehyde cross-linking resulted in a pronounced, concomitant effect on FN biochemical properties. Studies that include FN cross-linking or chemical coupling to the substrate should therefore investigate effects on both the physical and chemical properties of FN. FN conformation and adsorption strength also depend on the physicochemical properties of the substrate^78–80^. Our work suggests that the selection of underlying substrate could additionally regulate directional persistence in migration through modulating accessibility to its different domains, in the same way it impacts cell spreading^79^, proliferation and differentiation^81^. Moreover, our previous study concluding an increase of directional persistence as a function of substrate stiffness utilized covalently-coupled FN on hydrogels^82^, and thus could have underestimated the directional persistence of cells. Interestingly, the effect of substrate stiffness on FN fibrillogenesis is lost when FN is denatured^83^, highlighting the importance of combined substrate mechanics and FN conformation on mechanosensing as well.

FN fibrillogenesis depended on the nature of the substrate coating, and its inhibition reduced, at least in part, directional persistence in migration (Fig. 7). It is tempting to hypothesize that impaired fibrillogenesis on FN9–10/12–14 and FN120&FN40 (Fig. 7A and B) is responsible for reduced directional persistence on these coatings compared to FN (Fig. 1B). In other words, appropriate presentation of CBD and Hep2 domains on a substrate that is additionally permissive to FN fibrillogenesis could be sufficient to promote directional persistence at levels equal to full-length FN. Inhibition of FN fibrillogenesis was previously shown to inhibit the kinetics of epithelial cell closure *in vitro* ^84^, supporting our view that the ability of cells to assemble FN fibrils is associated with cell migration.

The lack of fibroblast motility upon EDA-FN knock-down was surprising; previous work has showed both an increase in cell speed upon cellular FN depletion^85, 86^ and the elimination of motility^87^, similar to our findings. Besides differences in cell type and experimental protocols, this discrepancy might result from the presence of an existing FN coating on the substrates in our case. The effects of silencing cellular FN and inhibiting protein synthesis on fibroblast motility are reminiscent of the cell migration defects observed upon inhibition of exosome-mediated secretion of fibronectin^88^. According to this model, exosomes provide necessary cell adhesion sites for migration through locally deposited FN, and consequently exosomes were dispensable for directional migration on FN pre-coated substrates^88, 89^. In our study, however, fibroblast motility was blocked even in presence of a FN coating under the cells, suggesting that knockdown of cellular FN blocks motility through intracrine signaling events or regulation of intracellular trafficking, and not by providing FN ligands. Importantly, a recent study identified a novel integrin endo/exocytosis pathway, which coupled integrin trafficking with polarized secretion of newly synthesized EDA-FN and FN fibrillogenesis^90^. Given the role of integrin trafficking in replenishing integrins at the leading edge to enable directional migration^91, 92^, it is plausible that EDA-FN synthesis and secretion are necessary for this process to occur.

Our efforts to identify kinases and associated intracellular pathways required for directional migration led to the surprising finding that cell speed and directional persistence were not correlated. Thus, our results suggest that the mechanisms responsible for regulating speed and directionality are distinct. Unexpectedly, inhibition of the FAK-Src-PI3K pathway did not reduce directional persistence, despite its central role in regulating lamellipodia protrusion^93^. This is in line with recent work supporting the view that PI3K is important in stabilization, rather than formation of new protrusions and chemotaxis^2, 61^. Interestingly, a slight increase of directional persistence after PI3K inhibition might reflect the absence of stabilization of randomly-formed, new protrusions, as previously suggested^62^. PKA inhibition downstream of integrin engagement was previously shown to impair polarization^26^ and modulate directional migration through VASP phosphorylation^94^. The lack of an effect upon PKA inhibition in our study suggests compensatory mechanisms in migration regulation under our experimental conditions, or off-target effects when high inhibitor concentrations were previously used. Our results confirmed the central role of the ERK-MAPK pathway in cell speed regulation^63, 65^, but did not support a role in regulating directionality. ERK-MAPK signaling modulates cell speed through control over lamellipodium protrusion dynamics^95, 96^, which we previously showed not to be correlated with high directional persistence of the whole cell over long time periods^4^.

In summary, the ability of full-length FN to induce directional migration on homogeneously-coated substrates requires integrin binding to the CBD as well as binding sites and/or signals stemming from the C-terminal Hep2 domain. While our reductionist system makes direct correlations with the complex *in vivo* situation difficult, several lines of evidence support the hypothesis that not merely FN deposition, but also its conformation are crucial during cell migration in 3D and *in vivo* ^11^. For example, full-length FN, but not FN fragments, was essential for directional migration^97^ and formation of complex micro-tissues^97, 98^ on engineered collagen gels. Also, FN-depleted clots were not permissive to fibroblast invasion, which was rescued when FN was co-polymerized with fibrin, but not when it was simply adsorbed on the clot^99^. Consequently, our observations should now be tested in more physiological setups, such as cell-derived matrices, in order to establish whether altered FN presentation has similar effects and potentially a physiological significance *in vivo*, where abnormal FN deposition^100, 101^ could alter the ability of cell to migrate with high directionality.

## Materials and Methods

### Materials and reagents

Reagents and antibodies used in this study are presented in Tables S1 and S2. CS1 peptide (sequence: DELPQLVTLPHPNLHGPEILDVPST) and a peptide with a scrambled sequence (GDPELNITLSVPLPTHLQEPDPVLH) were a gift from Prof. Inaam Nakchbandi (Max-Planck-Institute for Biochemistry, Martinsried, Germany). Recombinant fragments comprising the 12th-14th type three repeats of FN (Hep2) and the 9th-10th fused to the 12th-14th type three repeats of FN (FN9–10/12–14) were a gift from Prof. Jeff Hubbell (University of Chicago). Integrin α_5_β_1_ and α_v_β_3_ selective ligands (Fig. S3E) were prepared as previously described^47^. Fluorescent fibronectin (FFN) was prepared using fibronectin lyophilized from amine-free buffer (Life Technologies #33010018) and an Alexa Fluor 568 protein labeling kit (Life Technologies #A10238) or an Alexa Fluor 488 protein labeling kit (Life Technologies #A10235). FN-depleted fetal bovine serum (FBS) was prepared using gelatin sepharose-4B (GE Healthcare) column chromatography.

### Coatings

Substrates were coated overnight at 4 °C with PBS solutions of FN or FN fragments. For coating two fragments, these were mixed in solution prior to surface application. Optionally, FN-coated substrates were treated with glutaraldehyde or 4% paraformaldehyde (PFA) for 15 minutes at room temperature, or ice-cold MeOH for 5 minutes. Substrates were blocked with 1% BSA for 15 minutes at 37 °C, unless noted otherwise. The amount of adsorbed FN in 96-well plates (Greiner Bio-one) was quantified using a micro BCA protein assay kit (ThermoFisher Scientific); in this case, substrates were not blocked with BSA. Relative coating efficiencies were measured using a modified ELISA assay on 96-well plates as previously described^4^.

### Cell culture

The rat fibroblast cell line REF52 (REF)^4^ was cultured as a sub-confluent monolayer in Dulbecco’s modified eagle’s medium (DMEM), supplemented with 10% FBS and 1% penicillin/streptomycin (P/S). 3T3-FN fibroblasts^102^ were cultured in DMEM supplemented with 10% fetal calf serum (FCS) and 1% P/S. Cell lines were mycoplasma-free (tested monthly) and kept at 37 °C and 5% CO_2_, in a humidified atmosphere.

### Cell Adhesion Assay

REF adhesion was evaluated by quantifying the number of cells firmly attached on coated substrates at determined time points after seeding. Overnight serum-starved REF were detached and kept in suspension under ice for 10 minutes, with or without integrin selective antagonists, prior to seeding in coated 96-well culture plates under serum-free conditions. After predetermined time points, wells were washed twice with ice-cold PBS, the liquid carefully aspirated and culture plates were placed at −80 °C overnight. Relative cell numbers were quantified using the Cyquant cell proliferation assay kit (Life Technologies).

### Silencing of endogenous fibronectin

The siRNA used were a gift from Prof. Inaam Nakchbandi (Max-Planck-Institute for Biochemistry, Martinsried, Germany) and were as previously published^103^. The constructs used were: EDA siRNA #1: 5′ siRNA: 5′-GGGUUCUGAGUACACAGUCAGUGUG- dTdT-3′, 3′siRNA: 5′-GUGUGACUGACACAUGAGUCUUGG-dTdT-3′; EDA siRNA #2: 5′ siRNA: 5′-UCAGUGUGGUUGCCUUGCACGAUGA-dTdT-3′, 3′siRNA: 5′-AGUAGCACGUUCCGUUGGUGUGACU-dTdT-3′. For transfection, REF were plated in 6-well plates and the next day transfected with Promofectin (Promokine) according to the instructions provided by the supplier. After 48 hours, REF were washed once with PBS and incubated for 1 hour with serum-free DMEM prior to seeding.

### Single Cell Motility Assay

Overnight serum-starved REF were plated on coated TCPS Petri dishes (Greiner Bio-one) or 48-well culture plates (Greiner Bio-one) in serum-free medium at a density of 1–2 × 10^3^ cells/cm^2^. After 30 minutes, non-adherent cells were removed by aspiration and supplemented medium was added. In the case of FN40-coated substrates, we waited longer (1 hour) before removing non-adherent cells and adding serum. Live cells were imaged using phase contrast, time-lapse microscopy at 37 °C, in presence of 5% CO_2_. Images were acquired every 10 minutes for 16 hours, starting 4 hours after cell plating. A Delta Vision (DV) system (Applied Precision Inc.) on an Olympus IX inverted microscope, equipped with a cooled CCD camera and a 10x/0.3 NA (Olympus) objective were used. Cell trajectories were obtained using the ‘manual tracking’ plugin of ImageJ software and monitoring the displacement of the nucleus in each frame. Cells that 1) remained within the field of view, 2) did not divide and 3) were viable, were analyzed (typically 20 cells for each independent experiment). Speed was calculated as the total path length divided by time, and directionality index as the ratio of the distance from the origin to the trajectory length. For inhibition studies, the corresponding molecules were added 1 hour before time-lapse imaging.

### Fluorescence and confocal microscopy

Immunofluorescence microscopy was performed on cells fixed with 4% paraformaldehyde in PBS for 15 minutes at room temperature. Membranes were permeabilized using Triton X-100 (0.1%), followed by blocking with 1% BSA. Primary antibodies (diluted 1:100 in 1% BSA) were incubated for 1–2 hours at room temperature or overnight at 4 °C. Cells were then washed and incubated with secondary Alexa Fluor®-labeled antibodies (Life Technologies; diluted 1:150 in 1% BSA) for 1 hour at room temperature. DAPI and TRITC-phalloidin were used to stain nuclei and filamentous actin (F-actin). Images were acquired on the DV system described above, using a 60x/1.4 NA oil-immersion objective (Olympus), or a Zeiss LSM 880 laser scanning confocal microscope using a 63x/1.4 NA oil-immersion objective (Zeiss) or a 20x/0.8 NA objective (Zeiss).

### Image analysis

Cell projected area and aspect ratio, defined as the ratio of the major to the minor axis of a fitted ellipse, were determined through image analysis of single phalloidin-stained cells using the ‘Cell Outliner’ plugin of ImageJ. Focal adhesion area was determined using a custom-written macro in ImageJ, as previously described^82^. An area threshold of 0.4 μm^2^ was set to exclude small focal complexes and noise.

### Western Blotting

Overnight serum-starved REF were trypsinized and 1–2 million cells seeded on coated Petri dishes (60 mm) in serum-supplemented medium. Cells kept in suspension on ice for 15 minutes served as controls. After 30 minutes, adherent cells were rinsed with PBS and lysed. Suspension cells were centrifuged and washed twice with PBS prior to lysis. Details on the lysis buffer, SDS-electrophoresis and western blotting were previously reported^4^. Membranes were imaged using an Amersham Imager 600 (GE Healthcare). The antibodies used are presented in the supplemental information (Table S2).

## Electronic supplementary material

Supplementary Information

Video 1
