# Supplementary material for: Fibronectin promotes directional persistence in fibroblast migration through interactions with both its cell-binding and heparin-binding domains
Source: Sci Rep. 2017 Jun 16;7:3711. doi: 10.1038/s41598-017-03701-0 (PMC5473823; doi:10.1038/s41598-017-03701-0)
Supplement: Supplementary file 1 — Supplementary Information [file 41598_2017_3701_MOESM1_ESM.pdf]

Fibronectin promotes directional persistence in fibroblast migration through interactions with both its cell-binding and heparin-binding domains

Dimitris Missirlis, Tamás Haraszti, Horst Kessler, Joachim P. Spatz

Supplemental Information

Supplemental Table S1. Materials used in our study

Reagent	Abbreviation	Supplier	Cat. No.
**Dimethyl sulfoxide**	DMSO	Sigma	D8418
**phalloidin-tetramethylrhodamine B isothiocyanate**	TRITC-Phalloidin	Sigma	P1951
**Glutaraldehyde**	**-**	Sigma	G7651
**PF-573228**	PF 28	Sigma	PZ0117
**LY-294,002 hydrochloride**	LY	Sigma	L9908
**Bovine serum albumin**	BSA	Sigma	A4161
**Accutase**	-	Sigma	A6964
**bovine plasma fibronectin**	FN	Sigma	F1141
**H89 di-hydrochloride**	H89	Santa Cruz Biotechnologies	sc-3537
**PD98059**	PD98	Cell Signaling	9900S
**Heparin**	-	Thermo-Fisher	BP2524
**Bosutinib**	-	Hycultec	HY-10158
**Fibronectin alpha chymotryptic fragments of 120 kDa**	FN120	Merck-Millipore	F1904
**Fibronectin alpha chymotryptic fragments of 40 kDa**	FN40	Merck-Millipore	F1903
**Bisindolylmaleimide I**	BIM-I	Merck-Millipore	203290
**Recombinant human syndecan 4 ectodomain**	rhSynd4	R&D systems	2918-SD

**Cycloheximide**	-	Sigma	
**Vitronectin**	VN	Thermo-Fisher	PHE0011
**4% paraformaldehyde**	PFA	Santa Cruz Biotechnologies	sc-281692

Supplemental Table S2. Antibodies used in our study

Antibody	Clone	Application/Dilution	Supplier	Cat. No.
**anti-integrin α_4**	HP2/1	Blocking studies/-	Merck-Millipore	MAB1383
**anti-FN**	P1H11	ELISA / 1:10000	Merck-Millipore	MAB1926
**anti-FN**	A32	ELISA / 1:5000<br>Western Blot /1:1000	Thermo-Fisher	CSI005-32-02
**anti-FN**	IST-3	ELISA / 1:5000	Sigma	F0791
**anti-FN**	10	ELISA / 1:10000	BD Biosciences	610078
**anti-FN**	FN12-8	ELISA / 1:10000	QED Biosciences	42040
**anti-FN**	polyclonal #1	ELISA / 1:5000	Chemicon	AB2033
**anti-FN**	polyclonal #2	ELISA / 1:10000	Chemicon	AB2047
**anti-FN**	polyclonal #3	ELISA / 1:10000	Santa Cruz Biotechnology	sc-6952
**anti-pY**	PY99	Immunofluorescence / 1:100	Santa Cruz Biotechnology	sc-7020
**anti-tensin 1**	polyclonal	Immunofluorescence / 1:100	Sigma	SAB4200283
**anti-EDA-FN**	DH1	Immunofluorescence / 1:100	Merck-Millipore	MAB1940
**anti-cortactin**	polyclonal	Immunofluorescence / 1:100	Santa Cruz Biotechnology	sc-11408
**anti-Paxillin**	165	Immunofluorescence / 1:100	BD Biosciences	610619
**anti-mouse IgG AlexaFluor488 conjugate**	polyclonal	Immunofluorescence / 1:150	Thermo-Fisher	A11001
**anti-rabbit IgG AlexaFluor647 conjugate**	polyclonal	Immunofluorescence / 1:150	Thermo-Fisher	A31571
**anti-FAK**	77/FAK	Western Blot / 1:1000	BD Biosciences	610087

**anti-pFAK (Y397)**	14/FAK(Y397)	Western Blot / 1:1000	BD Biosciences	611722
**anti-pFAK (Y397)**	polyclonal	Immunofluorescence / 1:100<br>Western Blot / 1:1000	Sigma	F7926
**anti-β-actin**	AC-74	Western Blot / 1:1000	Sigma	A2228
**anti-mouse HRP conjugate**	polyclonal	Western Blot / 1:2000	Santa Cruz Biotechnology	sc-2005
**anti-rabbit HRP conjugate**	polyclonal	Western Blot / 1:2000	Santa Cruz Biotechnology	sc-2004

Supplemental Figures

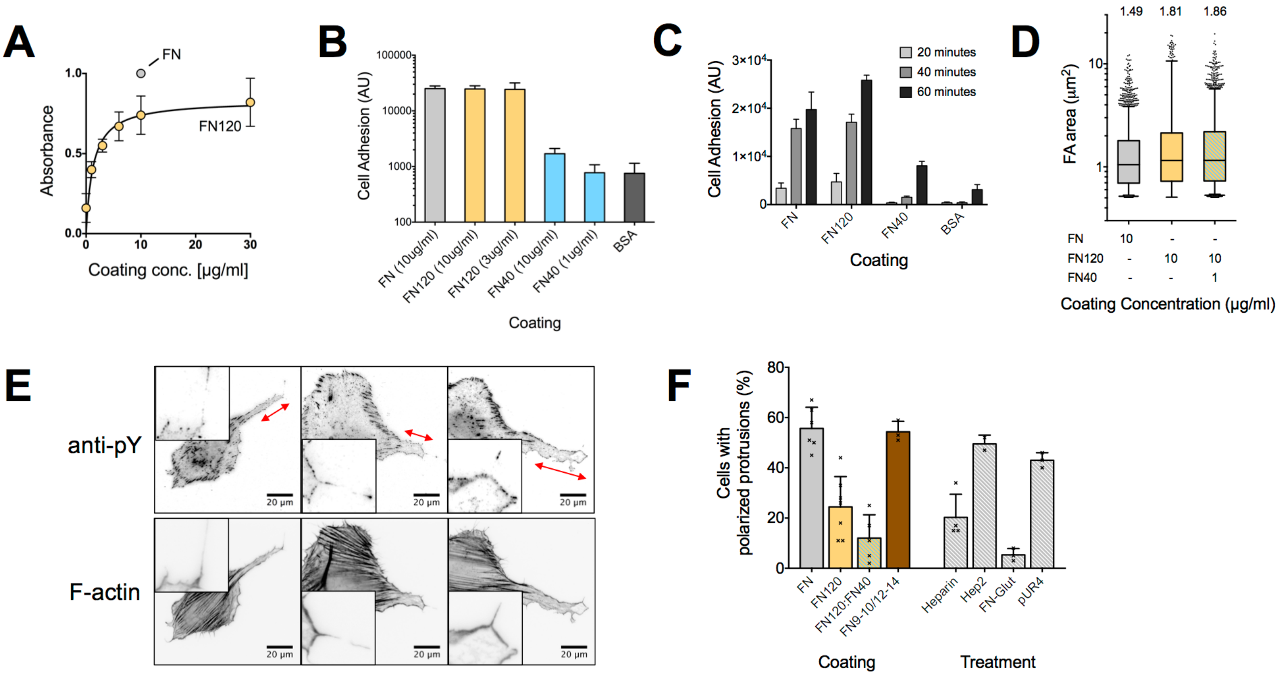

Figure S1. Characterization of fibroblast adhesion on FN and FN fragments. (**A**) Coating efficiency of adsorbed FN120 on TCPS, detected by an ELISA assay using a monoclonal anti-FN recognizing the cell-binding domain (clone P1H11). Surface saturation occurs at ≈10 µg/ml FN coating concentration. Data are normalized in respect to the signal obtained from a FN-coated substrate (10 µg/ml coating concentration) and are presented as mean±s.d. (n=3). Data are fitted with the equation **y**=A***x**/(B+**x**)+C. (**B**) REF adhesion on TCPS coated with FN or different FN fragments at the indicated coating concentrations. REF were incubated for 20 minutes with indicated substrates, washed 3 times with PBS and their numbers quantified using the Cyquant cell proliferation assay. Adhesion efficiency was similar between FN and FN120, and much lower for FN40. BSA-coated substrates (1% coating concentration) served as a negative control. Data from one (out of 3) independent experiment are presented as mean±s.d. (n=4). (**C**) REF adhesion as a function of incubation time on FN and FN fragments. REF were incubated for 20, 40 or 60 minutes with indicated substrates (10 µg/ml coating concentration) and their numbers quantified using the Cyquant cell proliferation assay. Data from one (out of 2) independent experiment are presented as mean±s.d. (n=4). REF adhesion increased monotonically on all coatings with time, but especially so for FN40. (**D**) The area of REF focal adhesions (FAs) on indicated coatings was quantified through immunofluorescence imaging of fixed cells 6 hours after seeding. The mean FA area (noted on the graph) is higher for REF on FN120 and FN120/FN40 coatings, compared to FN-coated substrates. The middle line in box plots indicates the median, the box indicates the interquartile range and the whiskers the 5th and 95th percentiles. (**E**) Examples of polarized protrusions on FN-coated glass

substrates (coating concentration: 10 μg/ml). Polarized protrusions are defined as protrusive regions of the cells that are decorated with nascent adhesions at their edge and that are free of large focal adhesions and stress fibers (indicated by red arrows). In most of cells, filopodia are present in the front of polarized protrusions (insets). Adhesions were identified by immunofluorescence against pY and F-actin was stained using phalloidin. (**F**) Percentage of REF exhibiting polarized protrusions on different substrates, or under different treatments. Coating concentrations of FN: 10 μg/ml, FN120: 10 μg/ml, FN120/FN40: 10/1 μg/ml and FN9-10/12-14 10 μg/ml. Concentration of soluble heparin: 100 μg/ml, Hep2: 30 μg/ml and pUR4 peptide: 10 μg/ml. Each data point (cross) represents an independent experiment, in which 40-100 cells were typically analyzed. Columns represent mean values and error bars standard deviations.

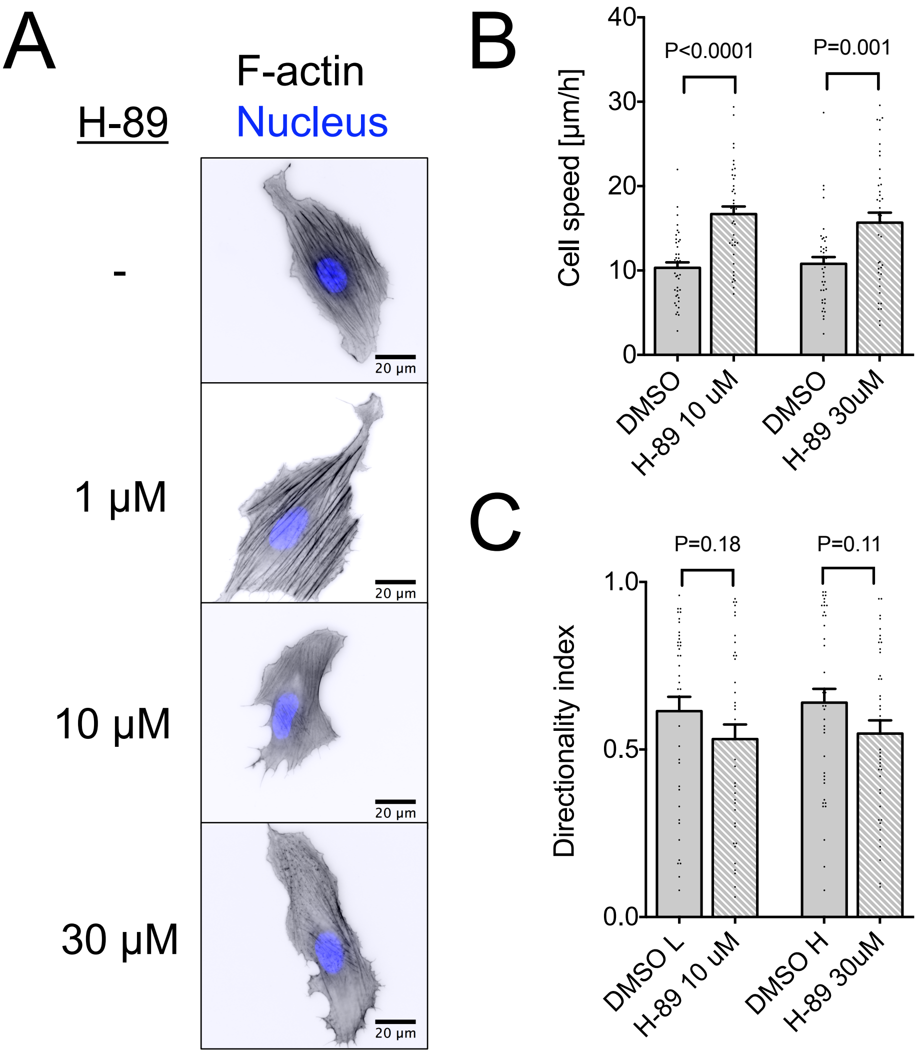

Figure S2. Incubation with high concentrations of the PKA small molecule inhibitor H89 affects cell migration and F-actin cytoskeleton organization of REF. (**A**) F-actin staining of REF seeded for 5 hours on FN-coated glass (coating concentration: 10 μg/ml) and further incubated for 1 hour with indicated concentrations of the PKA inhibitor H89. Above a concentration of 10 μM, loss of stress fibers was observed, consistent with reported inhibition of Rho-kinase by H89. Nuclei were stained with DAPI (blue). Scale bars: 20 μm. (**B**) REF speed and (**C**) directionality index calculated for REF on FN-coated TCPS (coating concentration: 10 μg/ml), in presence or absence of H89 at two different soluble concentrations. Experimental data in presence of inhibitor were compared to control conditions, where equal amounts of DMSO were added, using unpaired t-tests. Mean±s.e.m. are presented from 40 cells and 2 independent experiments.

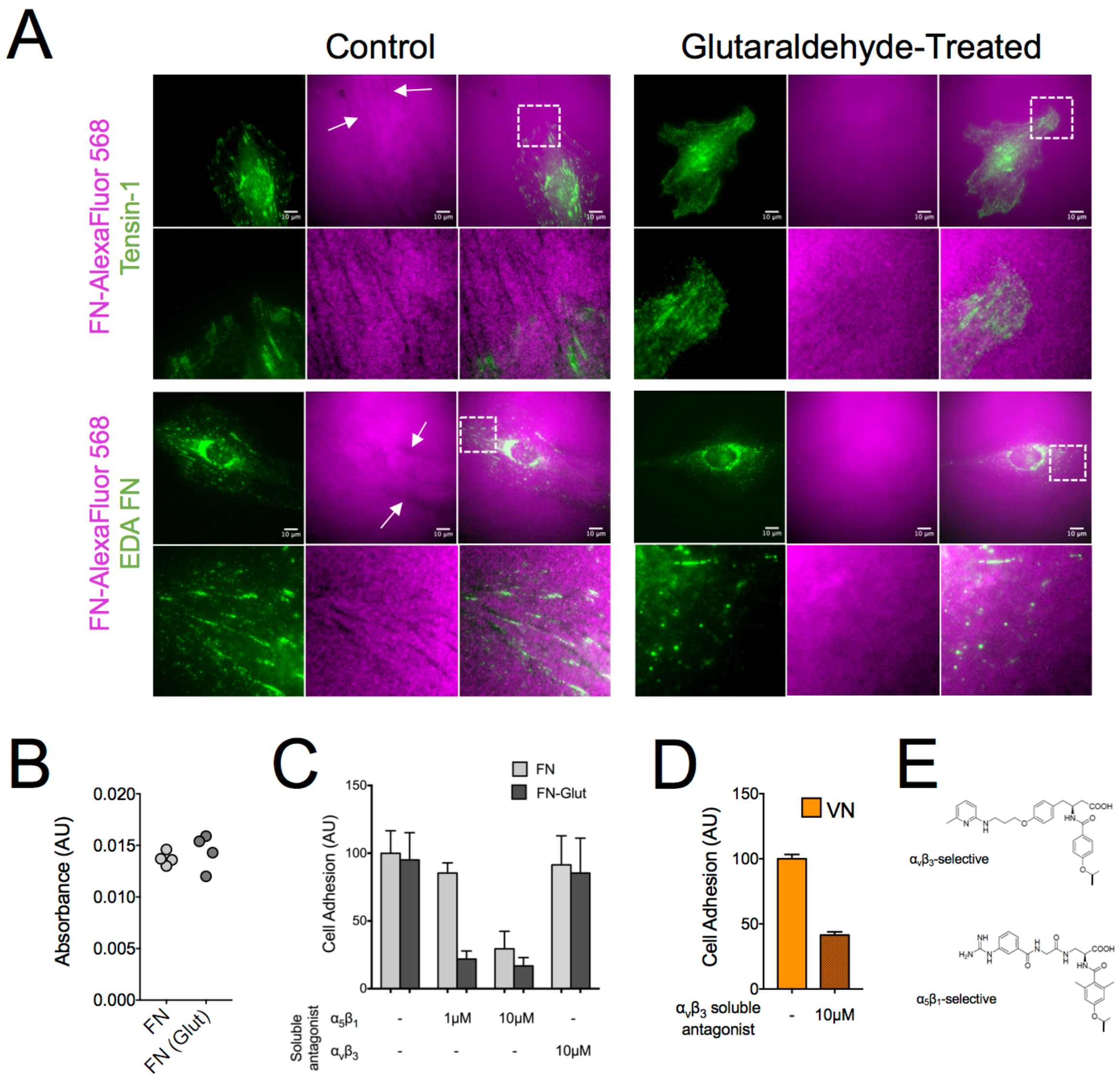

Figure S3. Glutaraldehyde cross-linking impairs fibrillar adhesion formation. (**A**) Glass substrates were coated with 10 µg/ml of fluorescently-labeled FN (Alexa Fluor 568; magenta) and some were further treated with glutaraldehyde to cross-link FN. REF were cultured for 24 hours on substrates, fixed and immunostained against tensin-1 or cellular FN (FN containing the alternatively spliced EDA domain). Fluorescence microscopy images revealed the absence of tensin- and cellular FN-containing fibrillar adhesions on cross-linked FN. Substrate adsorbed FN was removed in streak-like patterns under the cells in control conditions (white arrows), but not after glutaraldehyde cross-lining. Scale bars 10 µm. (**B**) The amount of adsorbed FN, with or without glutaraldehyde treatment was found to be equal using the micro-BCA assay. One (of two) independent experiment is presented. (**C**) Normalized REF adhesion to FN or FN-Glut (10 µg/ml coating concentration) 20 minutes after seeding, in presence of selective integrin antagonists against $\alpha_5\beta_1$ or $\alpha_v\beta_3$ are shown. Data are normalized to control conditions (FN) in the absence of integrin antagonists. The presence of $\alpha_5\beta_1$ integrin antagonists inhibited fibroblast adhesion on FN, whereas $\alpha_v\beta_3$ integrin antagonists did not. At 1 µM, the $\alpha_5\beta_1$ integrin antagonist blocked REF adhesion to FN-Glut but not FN, suggesting that

cells are more sensitive to $\alpha_5\beta_1$ blocking when adhering to FN-Glut. The mean±s.d. from 3 independent experiments are presented. (**D**) Normalized REF adhesion to VN-coated substrates (2 μg/ml coating concentration) 30 minutes after seeding. Soluble $\alpha_v\beta_3$ integrin antagonists inhibited REF adhesion to VN, confirming their efficacy. Mean±s.d from 4 measurements, and one of two independent experiments are shown. (**E**) Chemical structure of integrin-selective antagonists used for blocking integrins.

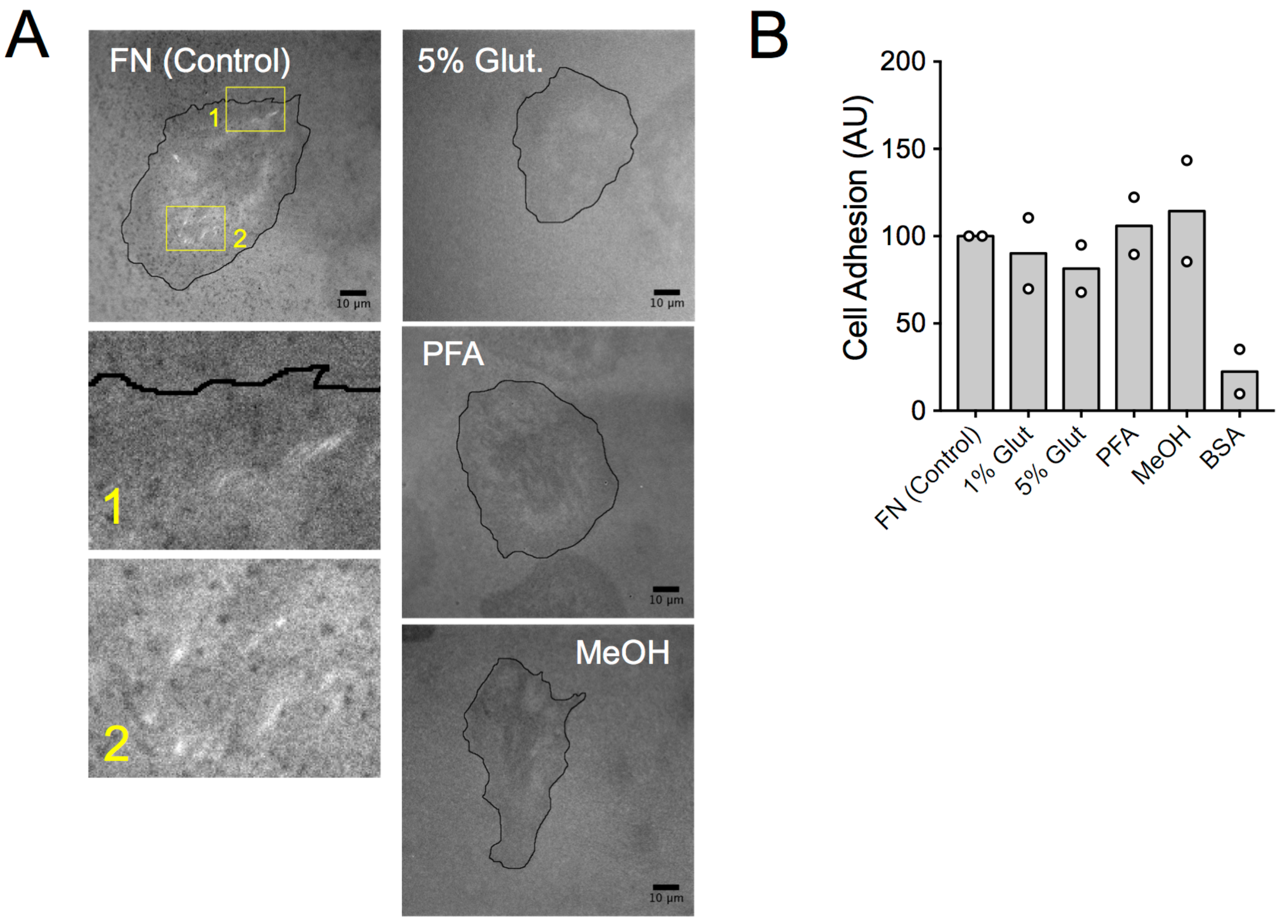

Figure S4. Application of different fixation agents on FN-coated substrates inhibits FN remodeling but does not impair cell adhesion. (**A**) Epifluorescence microscopy images of glass coverslips coated with fluorescently-labeled FN (Alexa Fluor 488) under live REF cells. FN-coated substrates were either non-treated (control) or treated with 5% glutaraldehyde for 15 minutes, 4% PFA for 15 minutes or ice-cold MeOH for 5 minutes. Images were acquired 4 hours after REF seeding on substrates. Cell outlines are drawn. Remodeling (fibrillogenesis) of adsorbed FN was observed only on control substrates. (**B**) Normalized REF adhesion on TCPS coated with FN, non-treated (control) or treated with indicated fixation agents. REF were incubated for 30 minutes with indicated substrates, washed 3 times with PBS and cell density was quantified using the Cyquant cell proliferation assay. Adhesion efficiency was similar between control and treated substrates. BSA-coated substrates (1% coating concentration) served as the negative control. The mean from two independent experiments are presented (n=5 samples for each independent experiment).

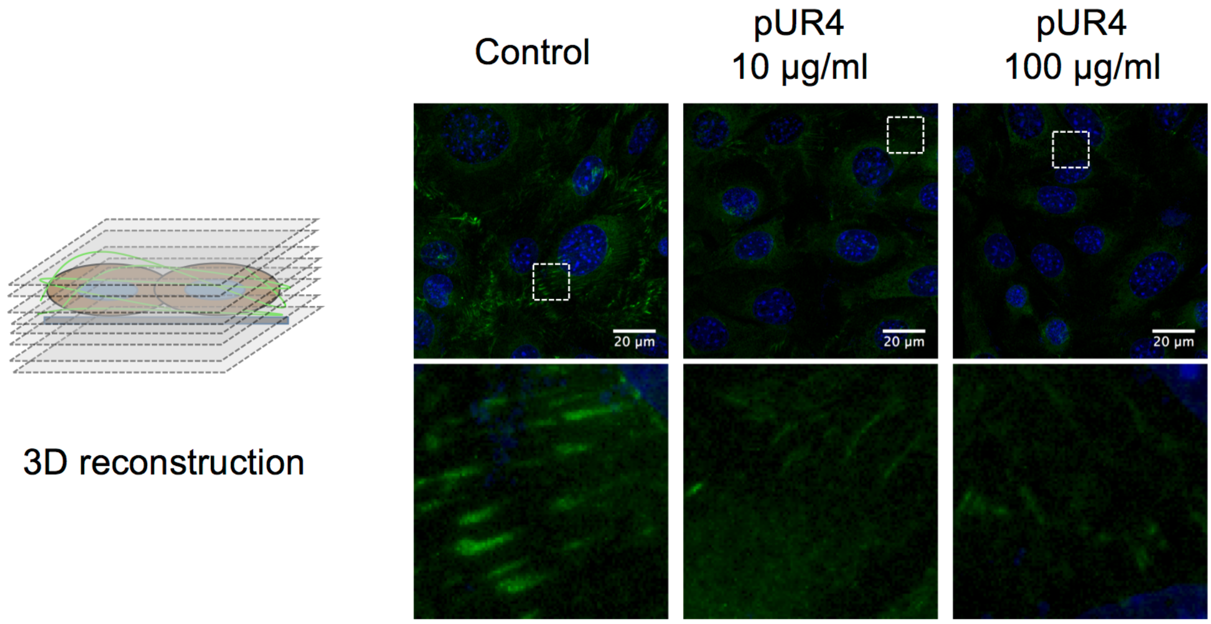

Figure S5. Inhibition of FN fibrillogenesis on the basal side of 3T3-FN fibroblasts by pUR4 peptide. Confocal microscopy 3D image reconstructions of secreted fluorescent FN (green) from 3T3-FN cells, 6 hours after seeding on FN-coated glass (10 µg/ml coating concentration), in absence or presence of indicated concentrations of the FN fibrillogenesis inhibitors pUR4. pUR4 inhibited FN fibril formation at both 10 and 100 µg/ml. Images were taken under the same illumination settings and normalized in respect to intensity. Bottom row shows zoomed-in regions, highlighted in the top row images. Nuclei are stained with DAPI (Blue). Scale bars: 20 µm.

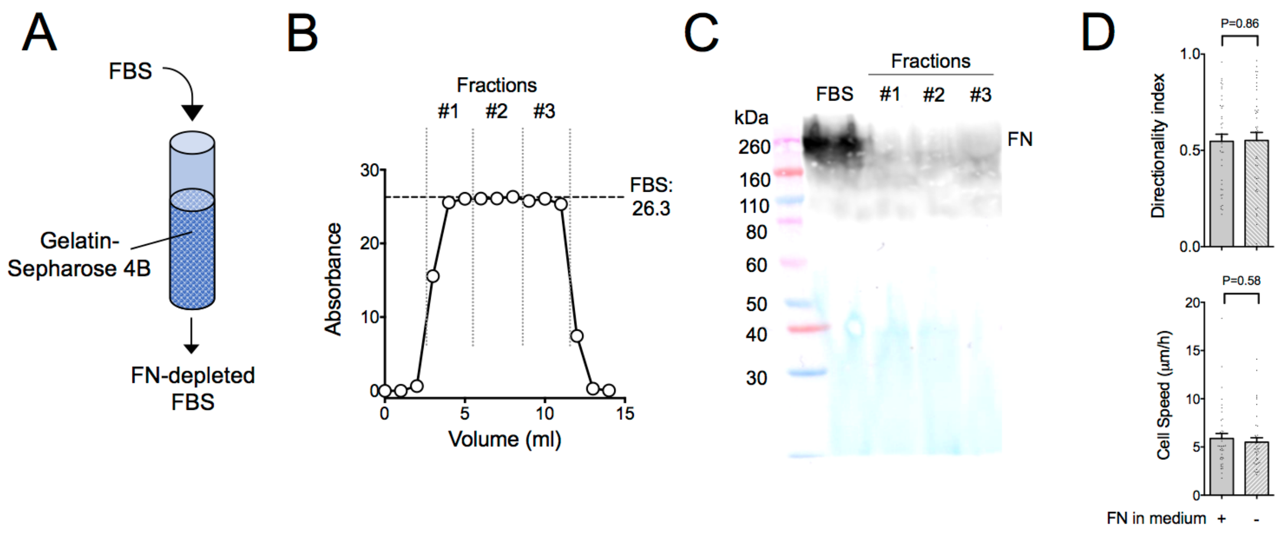

Figure S6. Directional persistence in fibroblast migration does not depend on the presence of soluble FN in the cell culture medium. (**A**) Schematic of the setup used to deplete FN from fetal bovine serum (FBS). FBS was run through a column packed with Sepharose 4B coupled to gelatin. (**B**) Chromatogram from 10 ml FBS applied on the column showing the absorbance (280 nm) of collected samples and prepared fractions. The absorbance of FBS is indicated on the graph. (**C**) Western blot analysis demonstrating the depletion of plasma FN in collected fractions. A monoclonal anti-FN antibody (A32) was used for detection. (**D**) REF speed and directionality index calculated for REF on FN-coated TCPS (coating concentration: 10 µg/ml), in presence of 10% FBS or 10% FBS depleted of FN. Data were compared using unpaired t-tests. Mean±s.e.m. are presented from 40 cells and 2 independent experiments.

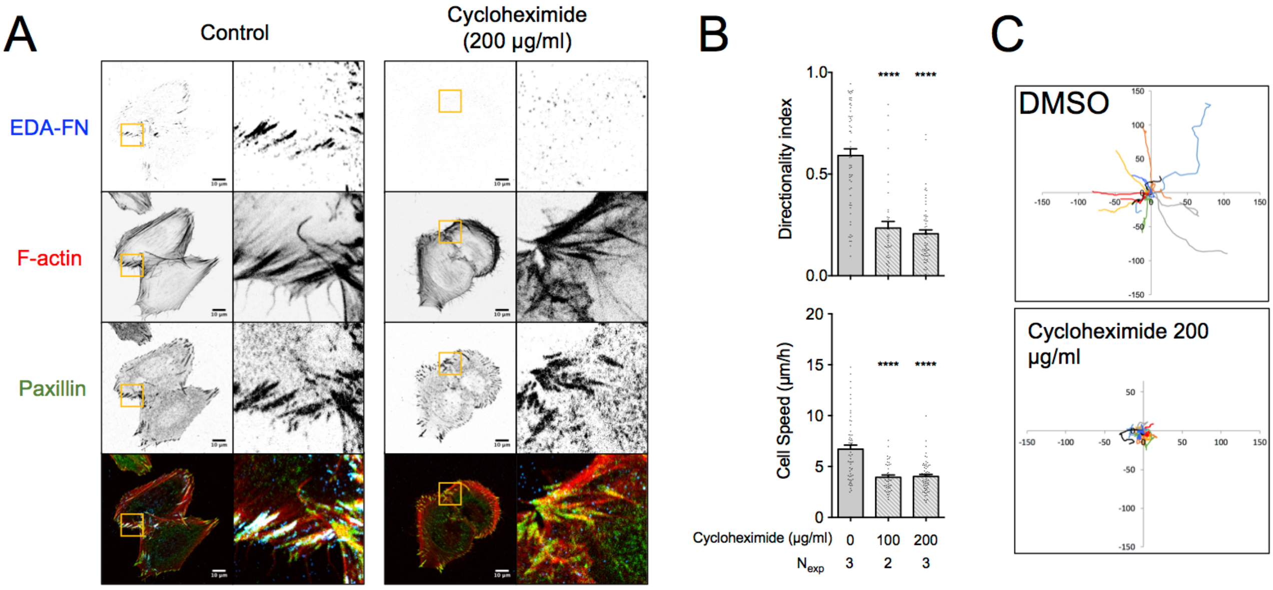

Figure S7. Inhibition of protein synthesis abolishes fibroblast migration. (**A**) Confocal microscopy images of REF treated with cycloheximide revealed lack of EDA-FN assembly on their basal side. The intensity of EDA-FN images was normalized. REF were seeded on FN-coated substrates (10 µg/ml coating concentration) in serum-free medium. After 1 hour, medium containing FBS with cycloheximide (200 µg/ml) or equivalent volume of DMSO was added, and cells were fixed 5 hours later before staining against indicated proteins. (**B**) REF speed and directionality index calculated for REF on FN-coated TCPS (coating concentration: 10 µg/ml), in FBS-supplemented medium and in the presence of cycloheximide or DMSO as the control. Data were compared using one way ANOVA analysis (**** $P<0.0001$). Mean±s.e.m. are presented. N_{exp}: number of independent experiments. (**C**) Random trajectories of REF in presence or absence of 200 µg/ml cycloheximide. In presence of cycloheximide, REF did not migrate away from their original position; trajectories reflect nuclei movements within the cell body.
